# Shoreline response to sea-level rise according to equilibrium beach profiles

**DOI:** 10.1038/s41598-023-42672-3

**Published:** 2023-09-22

**Authors:** Pau Luque, Lluís Gómez-Pujol, Francesca Ribas, Albert Falqués, Marta Marcos, Alejandro Orfila

**Affiliations:** 1 Mediterranean Institute for Advanced Studies (IMEDEA), Spanish National Research Council - University of the Balearic Islands (CSIC-UIB), Esporles, Spain; 2 Earth Sciences Research Group, Department of Biology, University of the Balearic Islands (UIB), Palma, Spain; 3 Department of Physics, Polytechnic University of Catalonia (UPC), Barcelona, Spain; 4 Department of Physics, University of the Balearic Islands (UIB), Palma, Spain

**Keywords:** Physical oceanography, Physical oceanography, Climate-change impacts

## Abstract

Shoreline position is a key parameter of a beach state, often used as a descriptor of the response of the system to changes in external forcing, such as sea-level rise. Changes in shoreline position are the result of coupled hydrodynamic and morphodynamic processes happening in the nearshore and acting at different temporal scales. Due to this complexity, methodologies aimed at reproducing shoreline evolution at decadal time scale require many simplifications. Simpler methods usually consider an equilibrium beach profile whose shape depends only on beach morphology, and whose location varies depending on incoming forcing. Here, we derive a general equation for shoreline evolution using equilibrium beach profiles. We particularize it based on several common assumptions, and evaluate changes on shoreline position caused by sea-level rise, combined with simultaneous wave and high-frequency sea-level forcing. We compare our model against other analytical equilibrium beach profile-based models and with a dynamic model explicitly computing sediment transport. Results indicate that: (i) it is necessary to consider the area of the emerged beach subject to marine forcing rather than focusing only on the submerged part, (ii) the rates of shoreline recession may change for narrow beaches, defined as those for which marine forcings act onto all of their aerial surface, and (iii) Bruun’s Rule can describe beach shoreline evolution, but the uncertainty in selecting the landward boundary of the active profile entails a huge uncertainty in the magnitude of shoreline evolution. This problematic uncertainty can be drastically reduced if instantaneous forcing conditions are used instead of the arbitrary emerged/submerged active profile boundaries typically defined by only one statistic parameter of extreme conditions.

## Introduction

Beaches are highly variable natural systems present along world coasts, formed by the accumulation of unconsolidated sediments shaped by the simultaneous action of waves, water levels, and currents^1,2^. The atmospheric and marine drivers mobilize the sediment thus reshaping the beach bed. This process occurs continuously, at different spatial and temporal scales, and presents feedback mechanisms that in turn affect future forcing and thus sediment mobilization^3–6^. The concept of equilibrium is often used, assuming stationary drivers that lead the system to a state of dynamic equilibrium in which acting forces are balanced, and where the distribution of bed elevations does not change over time^7^. However, for each instantaneous forcing conditions, an instantaneous theoretical equilibrium state can be defined, as the one that would be reached in the hypothetical case of those conditions remaining unchanged.

The changes in the beach bathymetry are intimately linked to changes in the shoreline position, as these are controlled by nearshore processes. Shoreline evolution is commonly described using shoreline orientation and cross-shore beach width. The former adjusts to the wave energy flux direction at the breaking point, while the latter responds to changes in the still-water level (${\overline{\eta }}$) and wave climate^8–10^. Beach bed transects in the cross-shore direction are called cross-shore profiles. They typically present a concave-upward shape in the region between the shoreline and the seaward limit where waves produce a significant sediment transport (defined by the depth of closure, $\hbox {h}_{\mathrm{c}}$). This shape varies in slope and width depending on the magnitude and frequency of incoming wave energy^11^. This pattern can be found in individual cross-shore transects, especially when alongshore sediment transport within the beach is negligible^12^. Generally, the pattern is easily identified in the alongshore-averaged cross-shore profile.

The equilibrium state for the alongshore-averaged cross-shore profile is referred to as Equilibrium Beach Profile (EBP) hereinafter. Because of its relative simplicity, EBP has been central in describing the long-term beach evolution, especially when the response of the beach to mean sea-level rise is concerned. There are numerous works that deal with the mathematical description of EBPs, either from theoretical considerations or from measurements^13^. However, EBP approaches have also been largely criticized arguing that their assumptions are poorly justified (e.g., Pilkey et al.^14^, and Cooper and Pilkey^15^). Despite this controversy, it is widely accepted that EBP assumptions are useful to describe the potential response of a beach to long-term forcings.

The use of EBP to study shoreline evolution was initiated by Bruun^16^, who considered that EBP responds to mean sea level ($\chi$) by moving without distortion. Specifically, he referred to the active part of an EBP (or active profile), i.e., the zone where natural cross-shore sediment transport occurs during a certain time period. According to Bruun^16^, the movement of the active profile induced by sea-level rise is composed of a vertical displacement equal to the change in mean sea level ($\Delta \chi$), plus a horizontal displacement required to provide the active profile with the sediment needed by the vertical displacement.

In a seminal work Bruun^16^ compared the position of two arbitrary EBP states, and assumed that the amount of sediment exchanged with and moved within the active profile corresponds to the changes in EBP topobathymetry arising from the active profile displacement. He considered an active profile of fixed size, defined by the extreme marine forcing conditions in the beach, i.e., those defined by the upper tail of the marine forcing climate (hereafter referred to as extreme conditions active profile), obtaining the so-called Bruun’s Rule:

1 $$\begin{aligned} \Delta \hbox {y}_{{\mathrm{s}},\chi } = - \frac{1}{{\widehat{\hbox {m}}}} \, \Delta \chi , \end{aligned}$$

where $\Delta \hbox {y}_{{\mathrm{s}},\chi }$ represents the change in EBP shoreline associated with changes in mean sea level ($\Delta \chi$), and where ${\widehat{\mathrm{m}}}$ is the slope of the active profile corresponding to the extreme conditions considered. In this way, Bruun^16^ introduced the idea that shoreline changes are proportional to changes in $\chi$, where the constant of proportionality between $\Delta \hbox {y}_{{\mathrm{s}},\chi }$ and $\Delta \chi$ is the ratio between the width and the height of the extreme conditions active profile.

When Bruun’s Rule is applied in the literature various locations for the active zone boundaries are used, meaning that different definitions for active profile width and active profile height are given. There is a generalized consensus that the offshore boundary of the active profile is given by a constant depth of closure (${\widehat{\mathrm{h}}}_{\mathrm{c}}$, also called shoreface depth^17,18^), characteristic of the upper tail of the beach wave climate, which defines the width of the submerged part of the active profile (${\widehat{\hbox {W}}}_{\mathrm{c}}$), spanning from the shoreline to the depth of closure. Equivalently, we can define the width (${\widehat{\mathrm{R}}}$) and height (${\widehat{\mathrm{B}}}$, relative to the shoreline elevation) of the extreme conditions active profile corresponding to the emerged part of the beach, so ${\widehat{\hbox {m}}} = ({\widehat{\hbox {B}}} + {\widehat{\hbox {h}}}_{\mathrm{c}}) / ({\widehat{\hbox {R}}} + {\widehat{\hbox {W}}}_{\mathrm{c}})$. However, the selection of the onshore boundary of the active profile is quite arbitrary, according to the current literature: it can be the dune crest^19^, the dune toe^20^, the berm height^21^, the landward limit of overwash deposition^22,23^, or even the shoreline^24^ (the latter corresponds to estimating the active profile slope using only its submerged part).

Again, there are different interpretations of berm behavior when using Bruun’s Rule: some authors claim it lifts with sea-level rise, and others state it remains at the same elevation. Other studies^13^ consider an unrealistic infinite slope at the shoreline by considering a discontinuity up to the berm height (${\widehat{\hbox {R}}} = 0$ but ${\widehat{\hbox {B}}} > 0$ in Eq. (1)). Whichever the interpretation, it is typically considered that Bruun’s Rule can only describe correctly berms whose vertical position changes, i.e. for the range of sea-level rise considered, are small compared to the berm height relative to the initial mean sea level^13^. Also, Eq. (1) is suitable only when shoreline recession is small compared to the ${\widehat{\hbox {W}}}_{\mathrm{c}}$^25^. Bruun’s Rule is generally considered an approximation, which can only provide an estimate of the shoreline recession due to mean sea level rise under quite restrictive conditions of applicability^15,26^ (and references therein), but due to its simplicity its use is widespread within the coastal research community^20^ and tends to be used for studies performed on a global scale^24^.

Following a similar reasoning, Edelman^27^ explicitly accounted for berms with fixed elevation, that is, whose height relative to the still-water level changes depending on this level. Instead of comparing two arbitrarily separated states, Edelman integrated the instantaneous velocity of the active profile displacement, which is equivalent to considering infinitesimal changes in the profile. By doing so, Edelman was able to obtain the following exact expression:

2 $$\begin{aligned} \Delta \hbox {y}_{\mathrm{s}} = - ({\widehat{\hbox {R}}} + {\widehat{\hbox {W}}}_{\mathrm{c}}) \,\,\, \ln \!\!\left( \frac{{\widehat{\hbox {h}}}_{\mathrm{c}} + {\widehat{\hbox {B}}} - {\overline{\eta }}(\hbox {t}_0)}{{\widehat{\hbox {h}}}_{\mathrm{c}} + {\widehat{\hbox {B}}} - {\overline{\eta }}(\hbox {t})}\right) , \end{aligned}$$

where $\hbox {t}_0$ is a reference time, ${\overline{\eta }}$ is the still-water level, and ${\widehat{\hbox {B}}}$ is the berm height with respect to the still-water level at time $\hbox {t}_0$.

In parallel, Dean^28^ derived an approximate expression to compute changes in EBP shoreline position caused by a change in sea still-water level and by the occurrence of wave setup, during an interval of constant breaking wave height:

3 $$\begin{aligned} \Delta \hbox {y}_{\mathrm{s}} = - \frac{\hbox {W}_{\mathrm{b}}}{{\widehat{\hbox {B}}} + \hbox {h}_{\mathrm{b}}} \left( {\overline{\eta }}(\hbox {t}) - {\overline{\eta }}(\hbox {t}_0)\right) - \frac{\hbox {W}_{\mathrm{b}}}{{\widehat{\hbox {B}}} + \hbox {h}_{\mathrm{b}}} \frac{7\gamma ^2}{80} \hbox {h}_{\mathrm{b}}, \end{aligned}$$

where ${\overline{\eta }}$ represents the still-water level, $\gamma = \hbox {H}_{\mathrm{b}} / \hbox {h}_{\mathrm{b}}$ is the breaker index (which is assumed to be constant in time and throughout the breaker zone), $\hbox {h}_{\mathrm{b}}$ is the breaking depth, referred to the level defined by the sum of the still-water level and wave setup (hereafter, time-averaged depth), and $\hbox {W}_{\mathrm{b}}$ is the width of the EBP between the shoreline and the breaking depth (i.e., assuming an infinite shoreline slope, presenting a discontinuity up to the berm height). This definition of the active profile width has been adopted by all the subsequent studies that apply Dean’s model^13,29,30^. In order to derive Eq. (3), Dean^28^ considered an elevation profile described by:

4 $$\begin{aligned} \hbox {z}(\hbox {y}) = \left\{ \begin{aligned}&{\widehat{\hbox {B}}}, \quad& \quad \hbox {y} < \hbox {y}_{\mathrm{s}} \\&{\overline{\eta }} + \zeta (\hbox {y} - \hbox {y}_{\mathrm{s}}) - \hbox {h}(\hbox {y} - \hbox {y}_{\mathrm{s}}), \quad& \quad \hbox {y} > \hbox {y}_{\mathrm{s}}&, \end{aligned} \right. \end{aligned}$$

where $\hbox {z}$ is the elevation of the EBP, $\hbox {y}$ is the cross-shore coordinate, $y_s$ is the cross-shore position of the EBP shoreline, $\zeta (\hbox {y}-\hbox {y}_{\mathrm{s}})$ is the wave setup (which varies along the profile), and $\hbox {h}(\hbox {y} - \hbox {y}_{\mathrm{s}})$ is the time-averaged depth. Dean^28^ also considered a specific shape for time-averaged depth: $\hbox {h}(\hbox {y} - \hbox {y}_{\mathrm{s}}) = \hbox {A} \, (\hbox {y} - \hbox {y}_{\mathrm{s}})^{3/2}$, where $\hbox {A} = 0.067 \, \hbox {w}_{\mathrm{s}}^{0.44}$, and $\hbox {w}_{\mathrm{s}}$ the sediment settling velocity. Notice, this shape is consistent with the assumption of an infinite shoreline slope (there is a discontinuity at the shoreline, where a finite jump to the berm height takes place).

Later, Miller and Dean^29^ stated that the generalization of Dean’s model (Eq. (3)) under time-varying wave conditions is:

5 $$\begin{aligned} \hbox {y}_{\mathrm{s}}(\hbox {t}) = \hbox {y}_{\mathrm{s}}(\hbox {t}_0) - \frac{\hbox {W}_{\mathrm{b}}(\hbox {t})}{{\widehat{\hbox {B}}} + \hbox {h}_{\mathrm{b}}(\hbox {t})} \left( {\overline{\eta }}(\hbox {t}) - {\overline{\eta }}(\hbox {t}_0) + \frac{7\gamma ^2}{80} \hbox {h}_{\mathrm{b}}(\hbox {t}) \right) . \end{aligned}$$

However, note that this equation implies that shoreline change between $\hbox {t}_0$ and $\hbox {t}$ depends on constant incoming wave conditions during the entire interval, corresponding to those occurring at $\hbox {t}$, and this applies to every time $\hbox {t}$. In other words, the variability and chronology of the wave forcing is completely neglected. The motivation to propose this generalization of Dean’s model, instead of just integrating Dean’s model for infinitesimal intervals of constant wave height is not justified in Miller and Dean’s text^29^.

Although Dean^28^ compared two arbitrarily separated EBP states to derive Eq. (3), later in the same article he used a continuity equation for sediment transport (referred to as sediment budget in that publication) to quantify how much sediment should be nourished to a beach in order to avoid the retreat of its shoreline caused by sea-level rise. Continuity equations have been used in other studies, generalizing that of sediment conservation in the active profile, usually as corrections to Bruun’s Rule to account for different specific sediment sources or sinks^31–33^. This generalized approach may be crucial for the correct analysis of the shoreline evolution associated to sea-level rise^34,35^. A very comprehensive work in the use of continuity equation of sediment transport and EBPs for shoreline evolution is the two-part article by Wolinsky^36^, and Wolinsky and Murray^22^. Specifically, Wolinsky^36^ derived a model by particularizing the integral version of Exner equation to the nearshore (Shoreline Exner Equation). This was later used by Wolinsky and Murray^22^ to assess the retreat of both a beach backed by a cliff and a barrier beach, up to timescales of thousands of years. Their conclusion was that shoreline retreat follows Bruun’s Rule (if the active profile is defined correctly) at a centennial scale, but at millenial timescales it ends following passive flooding, i.e., shoreline retreat following the inland slope. However, they did not consider the effects of wave setup on shoreline evolution, or how variable waves affect the active profile (especially its width), which is understandable given the long time scales explored.

Moreover, actual shoreline position may be different than that described by its equilibrium counterpart. In reality, changes in beach bed are not instantaneous, as they depend on sediment transport, which are quite difficult to model^37^. A strategy to circumvent this complexity is to use the concept of disequilibrium, which parameterize the tendency of a beach variable to approach its equilibrium counterpart^38^. For example, Miller and Dean^29^ based on laboratory and numerical experiments^39–41^, suggested that the actual beach shoreline tends to its EBP shoreline at an approximately exponential rate:

6 $$\begin{aligned} \frac{\hbox {dY}_{\mathrm{s}}}{{\mathrm{dt}}}(\hbox {t}) = - \hbox {k} \left( \hbox {Y}_{\mathrm{s}}(\hbox {t}) - \hbox {y}_{\mathrm{s}}(\hbox {t}) \right) , \end{aligned}$$

where $\hbox {Y}_{\mathrm{s}}$ is the instantaneous shoreline, $\hbox {y}_{\mathrm{s}}$ is the EBP shoreline, and $\hbox {k}$ is a time constant controlling the tendency of actual beach shoreline to the equilibrium one. They also proposed that $\hbox {k}$ should take different values for eroding or accreting shorelines.

Following similar ideas, there have been recent developments of several disequilibrium based shoreline evolution models, which are able to predict erosion and accretion cycles on hourly to decadal time scales^20^. More sophisticated models, e.g., Arriaga et al.^42^, are able to compute cross-shore sediment transport applying the disequilibrium concept to the whole bathymetric profile, as well as alongshore transport, thus allowing a more realistic bed evolution. In fact, this model has turned out to be significantly accurate to reproduce decadal coastal evolution^19,42,43^ and has been successfully applied to study the effects of sea-level rise on beach shoreline^19,21^.

In general, previous studies analyzing shoreline evolution using EBPs were vague when defining the cross-shore regions considered by their models, and some of them were inaccurate when describing the derivation and the integration of their equations, which may have caused misapplications of these models. Moreover, many authors did not derive mathematical expressions for changes in the shoreline caused by sea-level rise, and according to our research, nobody considered the effects a finite cross-shore size of the emerged beach. For this reason, we aim to: i) elucidate and unambiguously define which is the active zone that should be considered for EBP-based shoreline evolution models, ii) highlight inconsistencies found in some previous studies to avoid misinterpretation and misuse of these models, iii) derive analytical expressions for the effects of sea-level rise on shoreline evolution, according to different models, and iv) explore the effects of the emerged beach cross-shore size on shoreline evolution. We therefore present and discuss a general EBP shoreline evolution model, without any constraints in marine drivers or EBP shape, which sets the basis for further simplifications. We then particularize the model for the case of sediment conservation and an EBP shape depending only on instantaneous still-water level and on a stationary wave climate (statistically equivalent to historical data), which are assumptions commonly applied in the literature, and we solve it while considering the dependency on the emerged beach cross-shore size. We also propose analytical solutions for the impact of mean sea-level rise as described by Dean^28^ and Miller and Dean^29^. Our results of shoreline evolution are compared to those from the references above. We also use the results of Q2Dmorfo, considered to be a reasonable ground truth, to compare to our results of shoreline evolution under the assumptions of sediment conservation and dependence of EBP on instantaneous still-water level, when the beach is forced by mean sea-level rise.

It would be interesting to compare our findings with real data. However, we deliberately avoided this exercise for three reasons. First, our objective was to contextualize our results within the equilibrium beach profile (EBP) theory. To this end, Q2Dmorfo seems to be the best choice as a ground truth, since comparing the new models with real beach systems would mix the contextualization of these models within the EBP theory, with the comparison of the EBP theory itself with reality. Secondly, the latter comparison would require a longer and more complex approach, that should describe the several works dealing with the comparison and critical evaluation of EBP theory and models with respect to real beach systems, present the used study site and data, and discuss the findings derived from this additional comparison, which is not within the scope of the present work. Thirdly, any comparison to observational data would require a suitable study site, with a strong sea-level rise signal with respect to the storm surge and wave effects on shoreline evolution, for an observational period with a long time span.

## Methods

### New EBP models

In a general sense, EBP elevation can be modeled using a piecewise function. For the active beach profile, we use one interval describing the part of emerged beach with appreciable sediment transport caused directly by marine forcing ($\hbox {z}_{{\mathrm{wet}}}$), and one interval describing the submerged beach ($\hbox {z}_{\mathrm{sub}}$) from the shoreline up to the depth of closure. Also, in order to describe sediment exchanges at the boundaries of the active profile, we extend the domain and incorporate one interval accounting for the landward part of the emerged beach where direct marine forcing sediment transport is negligible ($\hbox {z}_{\mathrm{dry}}$), and one interval accounting for the offshore part of the beach ($\hbox {z}_{\mathrm{deep}}$):

7 $$\begin{aligned} \hbox {z}(\hbox {y})&= \left\{ \begin{aligned}&\hbox {z}_{\mathrm{dry}}(\hbox {y,t}),&\quad \hbox {y}_{\mathrm{L}} \;\;\;\,\,&< \hbox {y}{} & {} < \hbox {y}_{\mathrm{d}}(\hbox {t}) \\&\hbox {z}_{\mathrm{wet}}(\hbox {y,t}) = \hbox {z}_{\mathrm{wet,ref}}(\hbox {y,t}) + \hbox {Z}_{\mathrm{wet}}(\hbox {y} - \hbox {y}_{\mathrm{s}}(\hbox {t});  {\vec{\text{p}}}(\hbox {t})),&\quad \hbox {y}_{\mathrm{d}}(\hbox {t})&< \hbox {y}{} & {} < \hbox {y}_{\mathrm{s}}(\hbox {t}) \\&\hbox {z}_{\mathrm{sub}}(\hbox {y,t}) = \hbox {z}_{\mathrm{sub, ref}}(\hbox {y,t}) - \hbox {h}(\hbox {y} - \hbox {y}_{\mathrm{s}}(\hbox {t}); {\vec{\text{p}}}(\hbox {t})),&\quad \hbox {y}_{\mathrm{s}}(\hbox {t})&< \hbox {y}{} & {} < \hbox {y}_{\mathrm{c}}(\hbox {t}) \\&\hbox {z}_{\mathrm{deep}}(\hbox {y}),&\quad \hbox {y}_{\mathrm{c}}(\hbox {t})&< \hbox {y}, \end{aligned} \right. \end{aligned}$$

where $\hbox {y}_{\mathrm{L}}$ is the landward limit of the beach, which will be assumed as a solid contour (e.g., a cliff or a promenade), and thus regarded as constant hereafter. Also, $\hbox {y}_{\mathrm{d}} = \max \left( \hbox {y}_{\mathrm{s}} - \hbox {R}, \hbox {y}_{\mathrm{L}}\right)$ is the position of the boundary between the active emerged beach (the zone with significant sediment transport induced by the direct action of marine forcing) and the rest of the emerged beach, with $\hbox {R}$ representing the width of the emerged active profile, $\hbox {y}_{\mathrm{s}}$ is the position of the EBP shoreline, and $\hbox {y}_{\mathrm{c}} = \hbox {y}_{\mathrm{s}} + \hbox {W}_{\mathrm{c}}$ is the position of the depth of closure, with $\hbox {W}_{\mathrm{c}}$ being the width of the submerged active profile. Furthermore, $\hbox {z}_{\mathrm{wet,ref}}$ and $\hbox {z}_{\mathrm{sub,ref}}$ are reference levels (which can present spatial variability) for the emerged and submerged parts of the active profile, respectively; $\mathbf {\mathrm{p}}$ represents the set of parameters that may affect the shape of the active profile (which can change over time), mainly related to sediment properties and marine forcing, $\hbox {Z}_{\mathrm{wet}}$ is an elevation relative the emerged active profile elevation reference, and $\hbox {h}$ represents the depth relative to the reference level of the submerged active profile elevation.

**Figure 1:**
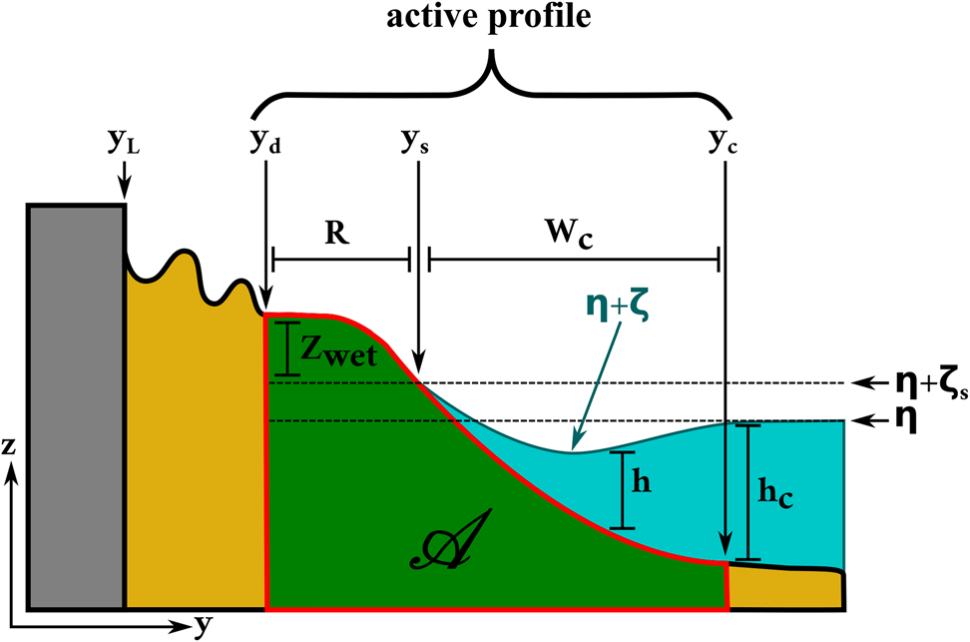
Proposed Fast EBP model for the wide beach case.

The width of the active profile is $\hbox {y}_{\mathrm{c}} - \hbox {y}_{\mathrm{d}} = \hbox {W}_{\mathrm{c}} + \min (\hbox {R,w})$, where $\hbox {w} = \hbox {y}_{\mathrm{s}} - \hbox {y}_{\mathrm{L}}$ is the width of the emerged beach. If the emerged beach width is large enough (wide beach case), two subregions are defined; if this is not the case (narrow beach case), only the emerged beach zone with appreciable sediment changes induced by marine forcing is considered. This EBP model and the different regions defined above are illustrated in Fig. 1.

Depending on the relationship between forcing and EBP response, different definitions for this piecewise function can be given. Here, we explore two different versions: i) referencing the EBP shoreline position to the instantaneous (including high-frequency) forcing, and describing its size and shape using also the instantaneous forcing, and ii) referencing the EBP shoreline position to the low-frequency component of instantaneous forcing, and describing its size and shape using the climatology of the forcing’s high-frequency content.

If the EBP shoreline is defined with respect to instantaneous forcing, meaning that the equilibrium beach bed adapts to instantaneous still-water level and instantaneous incoming waves, then $\hbox {z}_{\mathrm{wet, ref}} = {\overline{\eta }} + \zeta _{\mathrm{s}}$, and $\hbox {z}_{\mathrm{sub,ref}} = {\overline{\eta }} + \zeta (\hbox {y} - \hbox {y}_{\mathrm{s}}(\hbox {t}); \mathbf {\mathrm{p}}(\hbox {t}))$, where ${\overline{\eta }}$ is the still-water level, $\zeta$ is the wave setup, and $\zeta _s$ is the wave setup at the shoreline, and $\hbox {h}$ represents the time-averaged depth. This EBP model will be referred to as Fast EBP hereafter, and it is illustrated in Fig. 1.

If the EBP shoreline is defined with respect to the low-frequency component of marine forcing, meaning that it adjusts its position to long-term mean sea level and its shape to the climatology of waves and high-frequency components of the still-water level, then $\hbox {z}_{\mathrm{wet,ref}} = \chi$ and $\hbox {z}_{\mathrm{sub,ref}}=\chi$, referred to as Slow EBP model hereafter. In this case, time-averaged depth $\hbox {h}$ needs to be replaced by the elevation relative to mean sea-level (with its sign changed) ${\overline{\mathrm{h}}}$. It is important to note that ${\overline{\mathrm{h}}}$ is not a real depth, and so it can be negative. Moreover, due to the high-frequency variability in still-water level (oscillations around mean sea level), combined with the effects of incoming waves, the active profile will extend up to a distance characteristic of the upper tail of this high-frequency forcing. Therefore, we will substitute $\hbox {W}_{\mathrm{c}}$, $\hbox {R}$, $\hbox {z}_{\mathrm{wet}}(\hbox {y}_{\mathrm{d}})$, and ${\overline{\hbox {h}}}_{\mathrm{c}}$ by ${\widehat{\hbox {W}}}_{\mathrm{c}}$, ${\widehat{\hbox {R}}}$, ${\widehat{\hbox {B}}}$, and $\widehat{{\overline{\hbox {h}}}}_{\mathrm{c}}$, respectively, to indicate that the extreme conditions active profile is the one to be considered. Also, we will substitute $\hbox {w}$, by $w_\chi$, to indicate that we only get the low-frequency changes of emerged beach width, i.e., those associated to changes in mean sea level.

Notice that under both Fast EBP and Slow EBP formulations, the width and height of the emerged active profile may not correspond to the specific beach berm height and foreshore distance of the actual topography.

### Shoreline evolution equations

#### Fast EBP shoreline evolution equation

With the previous definitions, we can compute the active profile area between the beach bed elevation and a fixed datum (${\mathscr {A}}$ in Fig. 1). The time derivative of this area, which corresponds to the time derivative of the amount of sediment contained in the active profile according to the Fast EBP model, must be equal to the sum of the net flux of sediment entering the active profile and the sediment sources and sinks occurring within the active profile. Sediment fluxes through the cross-shore boundaries of the active profile can be further decomposed into those required by changes in the active profile position and size, and all the other ones (e.g., aeolian transport, river discharges). The corresponding EBP shoreline changes are given by (see Supplementary note 1):

8 $$\begin{aligned} \frac{\hbox {dy}_{\mathrm{s}}}{{\mathrm{dt}}}= & {} - \frac{\hbox {d}{\overline{\eta }}}{{\mathrm{dt}}} \, \frac{\min (\hbox {R, w}) + \hbox {W}_{\mathrm{c}}}{\hbox {z}_{{\mathrm{dry}}}(\hbox {y}_{\mathrm{d}}) - \hbox {z}_{\mathrm{deep}}(\hbox {y}_{\mathrm{c}})} - \left( \frac{\hbox {d}}{\hbox {dt}} \min (\hbox {R,w}) \right) \, \frac{\hbox {z}_{\mathrm{wet}}(\hbox {y}_{\mathrm{d}}) - \hbox {z}_{\mathrm{dry}}(\hbox {y}_{\mathrm{d}})}{\hbox {z}_{\mathrm{dry}}(\hbox {y}_{\mathrm{d}}) - \hbox {z}_{\mathrm{deep}}(\hbox {y}_{\mathrm{c}})} - \frac{\hbox {dW}_{\mathrm{c}}}{\hbox {dt}} \, \frac{\hbox {z}_{\mathrm{sub}}(\hbox {y}_{\mathrm{c}}) - \hbox {z}_{\mathrm{deep}}(\hbox {y}_{\mathrm{c}})}{\hbox {z}_{\mathrm{dry}}(\hbox {y}_{\mathrm{d}}) - \hbox {z}_{\mathrm{deep}}(\hbox {y}_{\mathrm{c}})} \nonumber \\{} & {} - \sum _{\mathrm{i}} \frac{\hbox {dp}_{\mathrm{i}}}{\hbox {dt}} \, \frac{\min (\hbox {R,w}) \, \frac{\partial \zeta _{\mathrm{s}}}{\partial \hbox {p}_{\mathrm{i}}} + \frac{\partial }{\partial \hbox {p}_{\mathrm{i}}} \int _{{\mathrm{y}}_{\mathrm{d}}}^{{\mathrm{y}}_{\mathrm{s}}} \hbox {Z}_{\mathrm{wet}} \; \hbox {dy} + \frac{\partial }{\partial \hbox {p}_{\mathrm{i}}} \int _{{\mathrm{y}}_{\mathrm{s}}}^{{\mathrm{y}}_{\mathrm{c}}} (\zeta - \hbox {h}) \; \hbox {dy}}{\hbox {z}_{\mathrm{dry}}(\hbox {y}_{\mathrm{d}}) - \hbox {z}_{\mathrm{deep}}(\hbox {y}_{\mathrm{c}})} + \frac{\hbox {Q}^\prime }{\hbox {z}_{\mathrm{dry}}(\hbox {y}_{\mathrm{d}}) - \hbox {z}_{\mathrm{deep}}(\hbox {y}_{\mathrm{c}})}, \end{aligned}$$

where $\hbox {Q}^{\prime }$ is an effective sediment flux that considers the net sediment flux entering from all the boundaries of the active profile plus the net effect of sediment sources and sinks (like nourishment’s, dredging, endogenous generation of sediment, *etc.*), minus the sediment fluxes required by changes in the active profile position and size. The first term of the RHS is related to vertical displacements of the active profile, the second and third terms relate to changes in the width of the active profile at the emerged beach and the submerged beach, respectively, the fourth term relates to changes in the active profile shape, and the fifth term relates to sediment fluxes, sources or sinks other than those generated by the change in the elevations of the active profile or its immediate surroundings.

Equation (8) is similar to the Shoreline Exner Equation of Wolinsky^36^. However, we also include the effects of wave setup and allow for discontinuities in the boundaries of the active profile. We do not consider any subsidence although it could be trivially considered by correcting the still-water level according to the total subsidence or lift since a reference time. Moreover, we regard instantaneous values for all the variables, both those corresponding to the forcing and those describing the profile size and shape. The complete derivation of Eq. (8) is given in the Supplementary Note 1.

If we assume that there are no sediment fluxes, sources, or sinks, other than those required by changes in the elevations of the active profile and its immediate surroundings, the last term in Eq. (8) becomes zero. In addition, assuming the equilibrium beach profile is continuous at the boundaries ($\hbox {z}_{\mathrm{dry}}(\hbox {y}_{\mathrm{d}}) = \hbox {z}_{\mathrm{wet}}(\hbox {y}_{\mathrm{d}})$ and $\hbox {z}_{\mathrm{sub}}(\hbox {y}_{\mathrm{c}}) = \hbox {z}_{\mathrm{deep}}(\hbox {y}_{\mathrm{c}})$),and also that $\hbox {Z}_{\mathrm{wet}}$ and $\hbox {h}$ have no dependence on time-varying parameters, Eq. (8) reduces to:

9 $$\begin{aligned} \frac{\hbox {dy}_{\mathrm{s}}}{{\mathrm{dt}}} = - \frac{\min (\hbox {R, w}) + \hbox {W}_{\mathrm{c}}}{\hbox {Z}_{\mathrm{wet}}(\hbox {y} = \hbox {y}_{\mathrm{d}}) + \zeta _{\mathrm{s}} + \hbox {h}_{\mathrm{c}} - \zeta _{\mathrm{c}}} \, \frac{\hbox {d}{\overline{\eta }}}{{\mathrm{dt}}} - \sum _{\mathrm{i}} \frac{\min (\hbox {R,w}) \, \frac{\partial \zeta _{\mathrm{s}}}{\partial \hbox {p}_{\mathrm{i}}} + \frac{\partial }{\partial \hbox {p}_{\mathrm{i}}} \int _{{\mathrm{y}}_{\mathrm{s}}}^{{\mathrm{y}}_{\mathrm{c}}} \zeta \; {\mathrm{dy}}}{\hbox {Z}_{\mathrm{wet}}(\hbox {y} = \hbox {y}_{\mathrm{d}}) + \zeta _{\mathrm{s}} + \hbox {h}_{\mathrm{c}} - \zeta _{\mathrm{c}}} \, \frac{\hbox {dp}_{\mathrm{i}}}{\hbox {dt}} . \end{aligned}$$

where we have substituted $\hbox {z}_{\mathrm{dry}}(\hbox {y}_{\mathrm{d}}) = \hbox {z}_{\mathrm{wet}}(\hbox {y}_{\mathrm{d}}) = \hbox {Z}_{\mathrm{wet}}(\hbox {y} = \hbox {y}_{\mathrm{d}}) + \zeta _{\mathrm{s}} + {\overline{\eta }}$ and $\hbox {z}_{\mathrm{deep}}(\hbox {y}_{\mathrm{c}}) = \hbox {z}_{\mathrm{sub}}(\hbox {y}_{\mathrm{c}}) = {\overline{\eta }} + \zeta _{\mathrm{c}} - \hbox {h}_{\mathrm{c}}$, and where $\zeta _{\mathrm{c}}$ represents the wave setup over the depth of closure.

At this point we remark that wave setup above depth of closure is small, and $\partial _{{\mathrm{p}}_{\mathrm{i}}} \int _{{\mathrm{y}}_{\mathrm{s}}}^{{\mathrm{y}}_{\mathrm{c}}} \zeta \; \hbox {dy}$ is also small, since the integral in the RHS tends to cancel out (wave setup presents positive values near the shoreline but negative values over the breaking point and the shoaling zone). Therefore, we can simplify the last equation to get:

10 $$\begin{aligned} \frac{\hbox {dy}_{\mathrm{s}}}{{\mathrm{dt}}} = - \frac{\min (\hbox {R,w}) + \hbox {W}_{\mathrm{c}}}{\hbox {Z}_{\mathrm{wet}}(\hbox {y} = \hbox {y}_{\mathrm{d}}) + \zeta _{\mathrm{s}} + \hbox {h}_{\mathrm{c}}} \, \frac{\hbox {d}{\overline{\eta }}}{{\mathrm{dt}}} - \sum _{\mathrm{i}} \frac{\min (\hbox {R,w})}{\hbox {Z}_{\mathrm{wet}}(\hbox {y} = \hbox {y}_{\mathrm{d}}) + \zeta _{\mathrm{s}} + \hbox {h}_{\mathrm{c}}} \, \frac{\partial \zeta _{\mathrm{s}}}{\partial \hbox {p}_{\mathrm{i}}} \, \frac{\hbox {dp}_{\mathrm{i}}}{{\mathrm{dt}}} . \end{aligned}$$

Although the equation is not linear, we can argue that high-frequency terms (storm surges, astronomical tides, wave setup...), only introduce oscillations of the emerged beach width around its low frequency evolution, which is controlled by sea-level rise. Thus, to analyze the effects of sea-level rise on beach width evolution, we can neglect the last term:

11 $$\begin{aligned} \frac{\hbox {dw}}{{\mathrm{dt}}} = - \frac{\min (\hbox {R,w}) + \hbox {W}_{\mathrm{c}}}{\hbox {Z}_{\mathrm{wet}}(\hbox {y} = \hbox {y}_{\mathrm{d}}) + \hbox {h}_{\mathrm{c}}^\prime } \, \frac{\hbox {d}{\overline{\eta }}}{{\mathrm{dt}}} , \end{aligned}$$

where $\hbox {h}_{\mathrm{c}}^\prime = \hbox {h}_{\mathrm{c}} + \zeta _{\mathrm{s}}$ is an effective depth of closure indicating the vertical dimension of the submerged beach active profile. At this point, it is important to recall the assumptions we included so far: i) the position of the landward extreme of the beach ($\hbox {y}_{\mathrm{L}}$) is fixed, and so we substituted $\hbox {dy}_{\mathrm{s}} / \hbox {dt}$ by $\hbox {dw} / \hbox {dt}$; ii) the equilibrium beach profile is continuous at the boundaries; iii) there are no sediment sources/sinks within the active profile; iv) the only sediment fluxes exchanged with the active profile are those required to displace it; and v) the shape of the EBP does not change with time. Therefore, the simplified version of our model can only be applied to beaches with a hard landward boundary, such as promenades or cliffs, and only for time scales for which their erosion is negligible. Moreover, elements outside the active profile can not actively exchange sediment with the active profile, so we are neglecting processes such as aeolian transport and overwash.

This simplified version of our model, Eq. (11), is comparable to Bruun’s Rule, Eq. (1). In order to handle it analytically and gain some knowledge of the system’s behavior, we perform a further step imposing that $\hbox {R}$ is constant over time (meaning that the emerged active zone width does not change with wave conditions) and that in some beaches it can be larger than the dry beach width $\hbox {w}$. Thereby, a clear separation arises between wide beach regime ($\hbox {w} > \hbox {R}$) and narrow beach regime ($\hbox {w} < \hbox {R}$).

To compare our model with that of Dean^28^, we need to repeat the previous derivation, but imposing that no sediment is mobilized by waves beyond the breaking point, i.e., considering $\hbox {h}_{\mathrm{b}}$ and $\hbox {W}_{\mathrm{b}}$ instead of $\hbox {h}_{\mathrm{c}}$, and $\hbox {W}_{\mathrm{c}}$, thus obtaining:

12 $$\begin{aligned} \frac{\hbox {dy}_{\mathrm{s}}}{\hbox {dt}} = - \frac{\min (\hbox {R,w}) + \hbox {W}_{\mathrm{b}}}{\hbox {Z}_{\mathrm{wet}}(\hbox {y} = \hbox {y}_{\mathrm{d}}) + \left( 1 + \frac{3\gamma ^2}{8} \right) \hbox {h}_{\mathrm{b}}} \, \frac{\hbox {d}{\overline{\eta }}}{{\mathrm{dt}}} - \frac{\min (\hbox {R,w}) + \hbox {W}_{\mathrm{b}}}{\hbox {Z}_{{\mathrm{wet}}}(\hbox {y} = \hbox {y}_{\mathrm{d}}) + \left( 1 + \frac{3\gamma ^2}{8} \right) \hbox {h}_{\mathrm{b}}} \, \frac{5\gamma ^2}{16} \, \frac{\hbox {dh}_{\mathrm{b}}}{{\mathrm{dt}}}, \end{aligned}$$

where $\zeta _{\mathrm{b}}$ is the wave setup at the breaking point. We use Bowen’s parametrization for wave setup^44^ (the same than Dean^28^ used): $\zeta _{\mathrm{b}} \approx - \hbox {h}_{\mathrm{b}} \gamma ^2 / 16$, and $\zeta = \zeta _{\mathrm{b}} + \frac{3 \gamma ^2}{8} \, \left( \hbox {h}_{\mathrm{b}} - \hbox {h} \right) \approx \frac{5 \gamma ^2}{16} \hbox {h}_{\mathrm{b}} - \frac{3 \gamma ^2}{8} \hbox {h}$.

There are two main differences between Eq. (12) and the original Dean’s model (Eq. (3)). First, Dean’s model neglects the dry beach width. Second, it does not take into account the amount of sediment involved in lifting or lowering the part of the emerged beach corresponding to the active profile (since it assumes it does not move vertically), which results in considering only the width of the submerged part of the active profile in the numerators of both terms. Third, Dean’s model implicitly assumes there is no wave setup in the initial state, which results in different coefficients multiplying $\hbox {h}_{\mathrm{b}}$ and $\frac{\hbox {dh}_{\mathrm{b}}}{\mathrm{dt}}$. We will not use Eq. (12) hereafter because the assumption behind Dean’s equations, that waves do not mobilize sediment between the breaking depth and the depth of closure, is not realistic. Instead, we will consider the more realistic Eq. (11) developed and we will compare it with the original Dean’s Eq. (3).

#### Slow EBP shoreline evolution equation

The derivation outlined in the previous section can be mimicked to describe Slow EBP shoreline evolution:

13 $$\begin{aligned} \frac{\hbox {dw}_\chi }{\hbox {dt}}= & {} - \frac{\hbox {d}\chi }{\hbox {dt}} \, \frac{\min ({\widehat{\hbox {R}}}, \hbox {w}_\chi ) + {\widehat{\hbox {W}}}_{\mathrm{c}}}{\hbox {z}_{\mathrm{dry}}(\hbox {y}_{\mathrm{d}}) - \hbox {z}_{{\mathrm{deep}}}(\hbox {y}_{\mathrm{c}})} - \left( \frac{\hbox {d}}{\hbox {dt}} \min ({\widehat{\hbox {R}}},\hbox {w}_\chi ) \right) \, \frac{\hbox {z}_{\mathrm{wet}}(\hbox {y}_{\mathrm{d}}) - \hbox {z}_{\mathrm{dry}}(\hbox {y}_{\mathrm{d}})}{\hbox {z}_{\mathrm{dry}}(\hbox {y}_{\mathrm{d}}) - \hbox {z}_{\mathrm{deep}}(\hbox {y}_{\mathrm{c}})} - \frac{\hbox {d}{\widehat{\hbox {W}}}_{\mathrm{c}}}{\hbox {dt}} \, \frac{\hbox {z}_{\mathrm{sub}}(\hbox {y}_{\mathrm{c}}) - \hbox {z}_{\mathrm{deep}}(\hbox {y}_{\mathrm{c}})}{\hbox {z}_{\mathrm{dry}}(\hbox {y}_{\mathrm{d}}) - \hbox {z}_{\mathrm{deep}}(\hbox {y}_{\mathrm{c}})} \nonumber \\{} & {} - \sum _{\mathrm{i}} \frac{\hbox {dp}_{\mathrm{i}}}{\hbox {dt}} \, \frac{\frac{\partial }{\partial \hbox {p}_{\mathrm{i}}} \left( \int _{{\mathrm{y}}_{\mathrm{d}}}^{{\mathrm{y}}_{\mathrm{s}}} \hbox {Z}_{\mathrm{wet}} \; \hbox {dy} - \int _{{\mathrm{y}}_{\mathrm{s}}}^{{\mathrm{y}}_{\mathrm{c}}} \hbox {h} \; \hbox {dy}\right) }{\hbox {z}_{\mathrm{dry}}(\hbox {y}_{\mathrm{d}}) - \hbox {z}_{\mathrm{deep}}(\hbox {y}_{\mathrm{c}})} + \frac{\hbox {Q}^\prime }{\hbox {z}_{\mathrm{dry}}(\hbox {y}_{\mathrm{d}}) - \hbox {z}_{\mathrm{deep}}(\hbox {y}_{\mathrm{c}})}. \end{aligned}$$

Assuming again continuity in the active profile boundaries, that there are no sediment fluxes, sources or sinks, other than those required by changes in the elevations of the active profile and its immediate surroundings, and also that $\hbox {Z}_{\mathrm{wet}}$ and $\hbox {h}$ have no dependence on time-varying parameters, we get:

14 $$\begin{aligned} \frac{\hbox {dw}_\chi }{\mathrm{dt}} = - \frac{\min ({\widehat{\hbox {R}}}, \hbox {w}_\chi ) + {\widehat{\hbox {W}}}_{\mathrm{c}}}{\hbox {Z}_{\mathrm{wet}}(- \min ({\widehat{\hbox {R}}},\hbox {w}_\chi )) + \widehat{{\overline{\hbox {h}}}}_{\mathrm{c}}} \, \frac{\hbox {d}\chi }{\hbox {dt}}. \end{aligned}$$

where the assumptions listed in the previous section are required. This equation is similar to the Essential Bruun Rule described by Wolinsky and Murray^22^, although some distinctions arise upon closer examination. Our model requires a fixed landward beach limit, defines active profile sizes based on the potential reach of wave action, and accounts for a narrow beach case (where the emerged beach width is smaller that this range). In contrast, Wolinsky and Murray^22^ considered a movable landward beach limit, and the potential presence of an overwash deposition zone (which is part of the active profile), even a cliff. These distinctions can lead to qualitative and quantitative differences between these models results, and make them useful for different ranges of application. Our model is suitable to analyze beaches with a hard landward limit, if overwash and other processes exchanging sediment with the active profile can be neglected, facilitating climate change assessments over decadal time scales. For extended time frames (on the order of hundreds or thousands of years), or scenarios where our specific assumptions may not hold, models such as that proposed by Wolinsky and Murray^22^ should be considered.

### Analytical integration of the models

In the following, we obtain an analytical integration for the effects of sea-level rise on beach shoreline, according to the proposed models, Fast EBP (Eq. (11)) and Slow EBP (Eq. (14)), original Dean’s model (Eq. (3)), and Miller and Dean’s model (Eq. (6)). Hereafter, we will assume still-water level is the sum of mean sea level ($\chi$, which represent the low-frequency component of ${\overline{\eta }}$), astronomic tides ($\Gamma$, which represent the predictable high-frequency component of ${\overline{\eta }}$), and storm surges ($\Xi$, which represent the stochastic high-frequency component of ${\overline{\eta }}$).

#### Fast EBP model integration

Within the wide beach regime ($\hbox {w} > \hbox {R}$), $\hbox {Z}_{\mathrm{wet}}(\hbox {y} = \hbox {y}_{\mathrm{d}})$ is constant under the assumption of constant $\hbox {R}$. We define $\hbox {B}_{\mathrm{w}} = \hbox {Z}_{\mathrm{wet}}(-\hbox {R}, \mathbf {\mathrm{p}})$, so Eq. (11) reads:

15 $$\begin{aligned} \frac{\hbox {dw}}{\hbox {dt}} = - \frac{\hbox {W}_{\mathrm{c}}(\hbox {t}) + \hbox {R}}{\hbox {B}_{\mathrm{w}} + \hbox {h}_{\mathrm{c}}^\prime (\hbox {t})} \, \frac{\hbox {d}{\overline{\eta }}}{\hbox {dt}}. \end{aligned}$$

Since the model is linear, we can just analyze the component associated with sea-level rise, and write it in integral form as:

16 $$\begin{aligned} \hbox {w}_\chi (\hbox {t}) - \hbox {w}_\chi (\hbox {t}_0) = - \int _{{\mathrm{t}}_0}^{\mathrm{t}} \left( \frac{\hbox {W}_{\mathrm{c}}(\tau ) + \hbox {R}}{\hbox {B}_{\mathrm{w}} + \hbox {h}_{\mathrm{c}}^\prime (\tau )} \, \frac{\hbox {d}\chi }{\hbox {dt}}(\tau )\right) \; \hbox {d}\tau . \end{aligned}$$

Since changes in $\chi$ are slower than changes in $\hbox {h}_{\mathrm{c}}^\prime$ by several orders of magnitude, Eq. (16) can be simplified as:

17 $$\begin{aligned} \hbox {w}_\chi (\hbox {t}) - \hbox {w}_\chi (\hbox {t}_0) = - \sum _{\mathrm{i}} \frac{\hbox {d}\chi }{\hbox {dt}}\left( \frac{\hbox {t}_{\mathrm{i}} + \hbox {t}_{\mathrm{i}+1}}{2}\right) \int _{{\mathrm{t}}_{\mathrm{i}}}^{{\mathrm{t}}_{{\mathrm{i}}+1}} \frac{\hbox {W}_{\mathrm{c}}(\tau ) + \hbox {R}}{\hbox {B}_{\mathrm{w}} + \hbox {h}_{\mathrm{c}}^\prime (\tau )} \; \hbox {d}\tau , \end{aligned}$$

and assuming ergodicity and stationarity for the depth of closure, we get the following for long enough integration times:

18 $$\begin{aligned} \hbox {w}_\chi (\hbox {t}) - \hbox {w}_\chi (\hbox {t}_0) = - \frac{1}{\hbox {m}_{\mathrm{w}}} \sum _{\mathrm{i}} \frac{\hbox {d}\chi }{\hbox {dt}}\left( \frac{\hbox {t}_{\mathrm{i}} + \hbox {t}_{{\mathrm{i}}+1}}{2}\right) \, \left( \hbox {t}_{{\mathrm{i}}+1} - \hbox {t}_{\mathrm{i}} \right) = - \frac{1}{\hbox {m}_{\mathrm{w}}} \left( \chi (\hbox {t}) - \chi (\hbox {t}_0)\right) , \end{aligned}$$

where we defined $\hbox {m}_{\mathrm{w}} = 1 \, / \, \hbox {E} \left[ (\hbox {W}_{\mathrm{c}}(\hbox {t}) + \hbox {R}) / (\hbox {B}_{\mathrm{w}} + \hbox {h}_{\mathrm{c}}^\prime (\hbox {t}))\right]$, with $\hbox {E}[\cdot ]$ denoting the expected value (which in this case is equivalent to a temporal average according to the different incoming wave conditions). We call this method Fast-Wide Rule. Notice that the functional form of this method is equal to that of Bruun’s Rule, but instead of the slope of the extreme conditions active profile it uses the average slope of the active profile. That is, due to time varying incoming wave conditions the active profile slope will be sometimes steeper, and sometimes milder, but the net effect will be the one corresponding to this average slope.

Under the narrow beach regime ($\hbox {w} < \hbox {R}$), $\hbox {Z}_{\mathrm{wet}}(\hbox {y} = \hbox {y}_{\mathrm{d}})$ can no longer be considered constant, since the distance between the shoreline and the beach back changes continuously. Accordingly, we define $\hbox {B}_{\mathrm{n}}(\hbox {w}) = \hbox {Z}_{\mathrm{wet}}(-\hbox {w},  \vec{\text{p}})$. We can not obtain a closed analytical expression for an arbitrary $\hbox {B}_{\mathrm{n}}(\mathrm{w})$, but we can derive an easy solution method based on a look-up table. Assuming we can decouple the high-frequency and low-frequency components of all magnitudes, the latter being related to sea-level rise, and following the same development of the previous section (but using $\hbox {w}_\chi$ instead of R), we can write:

19 $$\begin{aligned} \frac{\hbox {d} \hbox {w}_\chi }{{\mathrm{dt}}} = - \hbox {E}\left[ \frac{\hbox {W}_{\mathrm{c}}(\hbox {t}) + \hbox {w}_\chi }{\hbox {B}_{\mathrm{n}} (\hbox {w}_\chi ) + \hbox {h}_{\mathrm{c}}^\prime (\hbox {t})} \right] \, \frac{\hbox {d}\chi }{{\mathrm{dt}}} = - \frac{1}{\hbox {m}_{\mathrm{n}}(\hbox {w})} \, {\dot{\chi }}, \end{aligned}$$

where $\hbox {m}_{\mathrm{n}}(\hbox {w}_\chi ) = 1 \, / \, \hbox {E} \left[ (\hbox {W}_{\mathrm{c}}(\hbox {t}) + \hbox {w}_\chi ) / (\hbox {B}_{\mathrm{n}}(\hbox {w}_\chi ) + \hbox {h}_{\mathrm{c}}^\prime (\hbox {t})) \right]$, i.e., for each emerged active profile width, we can define an average slope by averaging in time according to the different incoming wave conditions. The last equation can be integrated in the following way:

20 $$\begin{aligned} \hbox {F}(\hbox {w}_\chi ) \equiv - \int _{{\mathrm{w}}_0}^{{\mathrm{w}}_\chi } \hbox {m}_{\mathrm{n}}(\hbox {w}_\chi ) \; \hbox {dw}_\chi = \chi (\hbox {t}) - \chi (\hbox {t}_0) \rightarrow \hbox {w}_\chi = \hbox {F}^{-1}\left( \chi (\hbox {t}) - \chi (\hbox {t}_0) \right) . \end{aligned}$$

That is, the integral of $\hbox {m}_{\mathrm{n}}(\hbox {w}_\chi )$ with respect to actual emerged beach width ($\hbox {w}_\chi$) between the initial beach width ($\hbox {w}_0$) and an arbitrary width $\hbox {w}$ corresponds to the change in mean sea level $\chi - \chi _0$ that brings the beach to that width. By computing this integral for several beach width and sea-level rise pairs a look-up table is created, representing shoreline evolution. Note we can generalize this equation to describe both the wide and narrow beach regimes if we redefine $\hbox {m}_{\mathrm{n}}(\hbox {w}\chi ) = 1 \, / \, \hbox {E} \left[ (\min (\hbox {R}, \hbox {W}_{\mathrm{c}}(\hbox {t})) + \hbox {w}_\chi ) / (\hbox {B}_{\mathrm{n}}(\min (\hbox {R}, \hbox {w}_\chi )) + \hbox {h}_{\mathrm{c}}^\prime (\hbox {t})) \right]$. We call this solution Fast-Narrow Rule.

Moreover, we can compute a closed analytical solution for the hypothetical case of a narrow beach but a constant $\hbox {Z}_{\mathrm{wet}}(\hbox {y} = \hbox {y}_{\mathrm{d}})$, $\hbox {B}_{\mathrm{n}}$, in order to gain some insight about the system behavior during the narrow beach regime. In this case, Eq. (11) reads:

21 $$\begin{aligned} \frac{\hbox {dw}}{\hbox {dt}} = - \frac{\hbox {W}_{\mathrm{c}}(\hbox {t}) + \hbox {w}}{\hbox {B}_{\mathrm{n}} + \hbox {h}_{\mathrm{c}}^\prime (\hbox {t})} \, \frac{\hbox {d}{\overline{\eta }}}{{\mathrm{dt}}}. \end{aligned}$$

Under these conditions, we can not separate the effects of sea-level rise as a distinct component, but for this case we can define these effects as the difference in emerged beach width evolution between the integration of the model with a forcing containing sea-level rise and another without it. An analytical solution can be then found (see the details in Supplementary Note 2), which allows one to compute beach shoreline evolution using only the time series of $\chi$, and a set of constant parameters derived from the high-frequency forcing. The derivation shows that the emerged beach width changes induced by sea-level rise are controlled by $\kappa = \hbox {E} \left[ \hbox {d}\Xi /\hbox {dt} / (\hbox {B}_{\mathrm{n}} + \hbox {h}_{\mathrm{c}}^\prime (\hbox {t})) \right]$. If $\kappa$ is negative, the solution blows out. If $\kappa$ is positive, changes in beach width are almost proportional to changes in the first time derivative of sea-level rise. In both cases, the solution does not have physical sense, so these options are not explored further. If $\kappa$ is zero, the effects of sea-level rise are described by:

22 $$\begin{aligned} \Delta \hbox {w}_{\chi }(\hbox {t}) = - \varepsilon _- \, \left( \frac{\hbox {w}(\hbox {t}_0)}{\mu } + \frac{1}{\beta \, \hbox {m}_{\mathrm{eff}}}\right) \, \left( 1 - \hbox {e}^{-\beta (\chi ({\mathrm{t}}) - \chi ({\mathrm{t}}_0))} \right) , \end{aligned}$$

where $\hbox {m}_{\mathrm{eff}} = 1 \, / \, \hbox {E}[\hbox {W}_{\mathrm{c}}(\hbox {t}) / (\hbox {B}_{\mathrm{n}} + \hbox {h}_{\mathrm{c}}^\prime (\hbox {t}))]$, $\beta = \hbox {E}[ 1 / (\hbox {B}_{\mathrm{n}} + \hbox {h}_{\mathrm{c}}^\prime (\hbox {t}))]$, $\varepsilon _- = \hbox {E}\left[ \hbox {e}^{-\beta (\Gamma (\hbox {t}) - \Gamma (\hbox {t}_0))}\right]$ (where $\Gamma$ represents astronomical tides), and $\mu$ is a factor arising from the stochastic oscillations of the high-frequency components of the still-water level (see the Supplementary Note 2 for a detailed explanation about $\mu$). We will refer to this method as Fast-Exponential Rule.

#### Slow EBP model integration

The Slow EBP model, Eq. (14), can be integrated following the same reasoning used before for the Fast EBP model. Under the wide beach regime ($\hbox {w}_\chi > {\widehat{\hbox {R}}}$), the solution is described by Eq. (18), although the active profile slope in this case is computed using extreme conditions, i.e., $\hbox {m}_{\mathrm{w}} = (\hbox {B}({\widehat{\hbox {R}}}) + \widehat{{\overline{\hbox {h}}}}_{\mathrm{c}})\, / \, ({\widehat{\hbox {W}}}_{\mathrm{c}} + {\widehat{\hbox {R}}})$. This will be referred to as Slow-Wide Rule hereafter. Under the narrow regime ($\hbox {w}_\chi < {\widehat{\hbox {R}}}$), the solution to the Slow EBP model presents the same form as the decoupled version defined above for the Fast EBP model, Eq. (19), which means its solution is described by Eq. (20), with $\hbox {m}_{\mathrm{n}}(\hbox {w}_\chi ) = ({\widehat{\hbox {B}}}(\hbox {w}_\chi ) + \widehat{{\overline{\hbox {h}}}}_{\mathrm{c}}) \, / \, ({\widehat{\hbox {W}}}_{\mathrm{c}} + \hbox {w}_\chi )$. Since the Slow-Wide Rule can be understood as a particular case of this solution, the shoreline evolution of a beach, independently of its width, can be computed just by building a look-up table, computed as indicated in Eq. (20), with $\hbox {m}_{\mathrm{n}}(\hbox {w}_\chi ) = ({\widehat{\hbox {B}}}(\min ({\widehat{\hbox {R}}}, \hbox {w}_\chi )) + \widehat{{\overline{\hbox {h}}}}_{\mathrm{c}}) \, / \, (\min ({\widehat{\hbox {R}}}, \hbox {w}_\chi ) + {\widehat{\hbox {W}}}_{\mathrm{c}})$. This will be called Slow-Narrow Rule hereafter.

In order to estimate the active profile for extreme conditions, a high quantile for both the highest elevation reached by waves in the emerged beach and the lowest elevation reached by waves in the submerged beach can be used. However, in order to account for the associated uncertainty, we propose to use a pair of quantiles defining a likely range that characterizes extreme conditions.

#### Dean’s model integration

According to Miller and Dean^29^, EBP shoreline changes associated with changes in mean sea level ($\chi$) are to be computed as (Eq. (5)):

23 $$\begin{aligned} \hbox {y}_{{\mathrm{s}},\chi }(\hbox {t}) - \hbox {y}_{{\mathrm{s}},\chi }(\hbox {t}_0) = - \frac{\hbox {W}_{\mathrm{b}}(\hbox {t})}{\hbox {B} + \hbox {h}_{\mathrm{b}}(\hbox {t})} \, \left( \chi (\hbox {t}) - \chi (\hbox {t}_{{0}})\right) , \end{aligned}$$

where $\hbox {t}_0$ is a reference time. We will refer to this equation as Raw Dean’s Rule hereafter. Note that this expression considers that, for the computation of EBP shoreline position for a time $\hbox {t}$, there is a constant breaking depth $\hbox {h}_{\mathrm{b}}$ (and thus a constant breaking wave height $\hbox {H}_{\mathrm{b}}$), during all the time interval between $\hbox {t}_0$ and $\hbox {t}$.

However, for time-varying wave conditions, Dean’s model (Eq. (3)) should be applied in a different way. Since it is meant for intervals of constant breaking wave height, it can be extended to time-varying wave conditions by considering infinitesimal intervals where $\hbox {h}_{\mathrm{b}}$ can be assumed constant. Considering only the component associated to sea-level rise (as in the section dealing with the Fast EBP model under the wide beach regime):

24 $$\begin{aligned} \hbox {y}_{{\mathrm{s}},\chi }(\hbox {t}) - \hbox {y}_{{\mathrm{s}},\chi }(\hbox {t}_0) = - \int _{{\mathrm{t}}_0}^{\mathrm{t}} \left( \frac{\hbox {W}_{\mathrm{b}}(\tau ) }{\hbox {B} + \hbox {h}_{\mathrm{b}}(\tau )} \frac{\hbox {d}\chi }{\mathrm{dt}}(\tau )\right) \; \hbox {d}\tau . \end{aligned}$$

This integral is solved following the same reasoning used before to derive Eq. (18) and yields:

25 $$\begin{aligned} \hbox {y}_{{\mathrm{s}},\chi }(\hbox {t}) - \hbox {y}_{{\mathrm{s}},\chi }(\hbox {t}_0) = - \frac{1}{\hbox {m}_{\mathrm{dean}}} \left( \chi (\hbox {t}) - \chi (\hbox {t}_0)\right) , \end{aligned}$$

where $\hbox {m}_{\mathrm{dean}} = 1 \, / \, \hbox {E} \left[ \hbox {W}_{\mathrm{b}}(\hbox {t}) / (\hbox {B} + \hbox {h}_{\mathrm{b}}(\hbox {t}))\right]$, the average slope of Dean’s EBP active profile. We name this solution as the Ergodic Dean’s Rule.

#### Miller and Dean’s model integration

Assuming that $\hbox {k}$ presents only one constant value, Eq. (6) can be written as^29^:

26 $$\begin{aligned} \hbox {Y}_{\mathrm{s}}(\hbox {t}) - \hbox {Y}_{\mathrm{s}}(\hbox {t}_0) = \int _{{\mathrm{t}}_0}^{\mathrm{t}} \hbox {k} \hbox {e}^{-{\mathrm{k}} (\hbox {t} - \tau )} \hbox {y}_{\mathrm{s}}(\tau ) \; \hbox {d}\tau , \end{aligned}$$

where $\hbox {Y}_{\mathrm{s}}$ represents the actual beach shoreline, and $\hbox {y}_{\mathrm{s}}$ is the associated EBP shoreline. We can find an analytical solution considering the expected value of this equation. We will take into account only the component associated to sea-level rise:

27 $$\begin{aligned} \hbox {Y}_{{\mathrm{s}}, \chi }(\hbox {t}) = \hbox {E}\left[ \int _{{\mathrm{t}}_0}^{\mathrm{t}} \hbox {k} \hbox {e}^{-{\mathrm{k}} (\hbox {t} - \tau )} \hbox {y}_{{\mathrm{s}},\chi }(\tau ) \; \hbox {d}\tau \right] = \int _{{\mathrm{t}}_0}^{\mathrm{t}} \hbox {k} \hbox {e}^{-{\mathrm{k}} ({\mathrm{t}} - \tau )} \hbox {E}\left[ \hbox {y}_{{\mathrm{s}},\chi }(\tau )\right] \; \hbox {d}\tau . \end{aligned}$$

The expected value of EBP shoreline is the same if we consider Raw Dean’s Rule or Ergodic Dean’s Rule, namely: $\hbox {y}_{\mathrm{s}}(\hbox {t}_0) - \, (\chi (\hbox {t}) - \chi (\hbox {t}_0)) / \hbox {m}_{\mathrm{dean}}$. Then, we can solve the resulting integral by parts:

28 $$\begin{aligned} \hbox {Y}_{{\mathrm{s}},\chi }(\hbox {t}) = - \frac{1}{\hbox {m}_{\mathrm{dean}}} \left( \chi (\hbox {t}) - \chi (\hbox {t}_0)\right) - \frac{1}{\hbox {m}_{\mathrm{dean}}} \sum _{\mathrm{n}=1}^\infty \frac{(-1)^{\mathrm{n}}}{{\mathrm{k}}^{\mathrm{n}}} \chi ^{({\mathrm{n}})}(\hbox {t}) + \hbox {e}^{-{\mathrm{k}} ({\mathrm{t}} - {\mathrm{t}}_0)} \left( \hbox {y}_{\mathrm{s}}(\hbox {t}_0) + \frac{1}{\hbox {m}_{\mathrm{dean}}} \sum _{{\mathrm{n}}=1}^\infty \frac{(-1)^{\mathrm{n}}}{{\mathrm{k}}^{\mathrm{n}}} \chi ^{({\mathrm{n}})}({\mathrm{t}}_0) \right) , \end{aligned}$$

where $\chi ^{(\mathrm{n})}(\hbox {t})$ represents the $\hbox {n}{\mathrm{th}}$ time derivative of sea-level rise, and where the last term is a transient (that becomes zero after approximately 3 - 5 times the time scale $\hbox {1/k}$). Notice that Eq. (28) presents Ergodic Dean’s Rule as the leading term, plus higher order corrections.

### Study case and Q2Dmorfo simulations

We simulated shoreline evolution in a synthetic beach inspired on Son Bou beach (Menorca Island, Western Mediterranean) for a period of 72 years, which was forced with coupled synthetic sea-level rise and high-frequency forcing from a hindcast. This site was selected because of the wealth of available observations. The Balearic Islands Observing and Forecasting System (SOCIB) has a continuous monitoring program since 2011 that includes, among other variables, biannual measurements of beach profiles and continuous wave and shoreline detection using ADCP and cameras, respectively^45^.

All models (including Q2Dmorfo) were forced with the same high-frequency wave and sea-level data, provided by Toomey et al.^46^. This hindcast data includes wave height, wave period and storm surge generated using a fully-coupled hydrodynamic and wave model, and we imposed shore-normal wave incidence (instead of the wave direction given by the hindcast). The data selected corresponds to a water depth of 30 m, so wave height data was propagated to the required depth by multiplying it with the shoaling coefficient between these two depths (i.e., assuming energy conservation of the wave train, proportional to the square of wave height, and also neglecting effects other than shoaling, such as reflection and breaking). Specifically, the wave height time series was first propagated to deep waters, then limited to its $99^{\mathrm{th}}$ percentile for computational purposes, and then propagated to the depth required by each case. Astronomic tides were computed using UTide^47^ to reconstruct the time series of the nearest tide gauge, located 20 km away inside Maó’s harbor. Sea-level rise was modeled as a parabolic curve reaching 1 m by the end of the simulation period of 72 years, to account for acceleration.

We set a displaced Dean EBP as the equilibrium profile, combined, in the emerged beach, with an exponential trend to a constant dry beach height, giving^42^:

29 $$\begin{aligned} \hbox {z}_{\mathrm{eq}}(\hbox {y,t}) = \left\{ \begin{aligned}&\hbox {z}(\hbox {y}_{\mathrm{s}},\hbox {t}) + \hbox {B}_\infty \, \left( 1 - \hbox {e}^{{\mathrm{m}}_{{\mathrm{s}}} \, \frac{\hbox {y} - \hbox {y}_{\mathrm{s}}(\hbox {t})}{\hbox {B}_\infty }} \right),&\quad \hbox {y} < \hbox {y}_{\mathrm{s}} \\&\hbox {z}(\hbox {y}_{\mathrm{s}},\hbox {t}) - \frac{3}{2} \, \hbox {m}_{{\mathrm{s}}} \, \hbox {y}_0^{1/3} \, \left( \left( \hbox {y} + \hbox {y}_0 - \hbox {y}_{\mathrm{s}}(\hbox {t}) \right) ^{2/3} - \hbox {y}_0^{2/3}\right),&\quad \hbox {y} > \hbox {y}_{\mathrm{s}}&, \end{aligned} \right. \end{aligned}$$

where $\hbox {m}_{\mathrm{s}}$ represents the slope of the profile at the shoreline, $\hbox {B}_\infty$ is the elevation of the landward extreme of the beach for an infinitely wide beach (relative to that of the shoreline), and $\hbox {y}_0$ is a parameter that is obtained fixing that the profile passes through a certain point (usually considering the elevation distribution of the submerged beach). The displaced Dean EBP used was calibrated using Son Bou measurements, obtaining $\hbox {y}_0 = 200\hbox { m}$, $\hbox {m}_{\mathrm{s}} = 0.035$, and $\hbox {B}_\infty = 2\hbox { m}$. However, the obtained EBP is not representative of the actual Son Bou beach, since it is constrained to a prescribed functional form. The most important source of discrepancy is the emerged beach width because the EBP is calibrated using only the shoreline slope and the backshore height of Son Bou beach (i.e., without considering the actual width of the emerged Son Bou beach).

In order to quantify the differences between the presented models and a common reference, we compared their shoreline evolution with that obtained using the Q2Dmorfo model^42^. Q2Dmorfo is a non-linear morphodynamic model for large scale coastal dynamics, which computes the wave field over the whole bathymetry and uses it to parameterize the depth-averaged sediment transport. Offshore wave conditions, assumed to be monochromatic, are applied at the seaward boundary of the computational domain, and propagated over the evolving bathymetry up to the breaking point using wave energy conservation and ray tracing. The sea-level time series is also imposed at the offshore boundary of the computational domain, and is assumed to be uniform throughout all the domain except in the surf zone, where a proxy for wave setup is introduced. The well-known CERC formula^48^ is applied for the alongshore transport, with local wave height and angle at breaking computed from the propagated wave field. The cross-shore transport is computed from the swash zone to the depth of closure, as proportional to the difference between the local bed slope and an EBP slope prescribed for that depth, while the proportionality constant depends on wave stirring. This approach causes a relaxation of the bathymetry to the EBP using the concept of disequilibrium. Moreover, alongshore diffusive transport is also assumed to represent bed level diffusion processes by wave action. Finally, the Exner equation is applied to compute the bed level evolution from the gradients of sediment transport.

For Q2Dmorfo simulations, the domain extended 400 m offshore from the initial shoreline in the cross-shore direction (up to about 11 m depth), and 100 m in the alongshore direction. The grid size was 2 m in the cross-shore direction, and 5 m in the alongshore direction, and the time step was 0.36 s. The parameter values for sediment transport were chosen following a Q2Dmorfo calibration performed in Cala Castell beach^43^ (Catalan coast, Western Mediterranean). However, the diffusivity factor was increased a factor of two because in this Q2Dmorfo application we only intend to perform an alongshore-uniform time evolution. The swash zone was assumed to have a wider extension, of 10 m, because Son Bou beach is more planar than Cala Castell. We set the displaced Dean EBP explained above as the equilibrium profile, an alongshore uniform initial topo-bathymetry equal to the equilibrium one, and zero sediment transport in the four model boundaries. This gave a Q2Dmorfo evolution equivalent to those simulated with the analytical models to be tested.

The topo-bathymetries obtained from Q2Dmorfo simulations were used to compute shoreline time series which were compared with the analytical model outputs. Specifically, Q2Dmorfo shoreline positions were estimated as the alongshore averaged Q2Dmorfo topo-bathymetry interpolated to the instantaneous mean sea level ($\chi$). We also interpolated the topo-bathymetry to the still-water level (${\overline{\eta }}$) with a 5-year min-max window to compute the instantaneous variability of shoreline position due to inundation.

The coefficients of all the presented analytical models were calibrated according to Toomey’s hindcast data^46^ and to the displaced Dean EBP described above. To estimate both the average conditions and extreme conditions active profile size, we require the instantaneous value of the highest elevation reached by waves with respect to mean sea level, which can be computed as the sum of storm surge, astronomical tide, and wave runup; as well as the instantaneous value of the lowest elevation reached by waves, which can be computed as the sum of storm surge, astronomical tide and depth of closure of that moment. In particular:

- For Bruun’s Rule: ${\widehat{\hbox {h}}}_{\mathrm{c}}$ was computed according to Birkemeier’s formula^49^, while ${\widehat{\hbox {W}}}_{\mathrm{c}}$ was computed inverting the calibrated displaced Dean profile at $\hbox {z}_{\mathrm{eq}}(\hbox {y}_{\mathrm{s}} + {\widehat{\hbox {W}}}_{\mathrm{c}},\hbox {t}) = \hbox {z}(\hbox {y}_{\mathrm{s}},\hbox {t}) - {\widehat{\hbox {h}}}_{\mathrm{c}}(\hbox {t})$. Due to the ambiguity in the selection of $\hbox {R}$, all possible values between 0 and $\hbox {w}_0$ were considered, while the corresponding $\hbox {B}$ for each case was computed following the emerged part of the calibrated displaced Dean profile. This means, the shoreline recession we computed for Bruun’s Rule is a range describing the possible outcome of this rule according to the ambiguity in the onshore active profile boundary selection.
- For Raw Dean’s Rule, ergodic Dean’s Rule, and Miller and Dean’s Rule: $\hbox {B} = \hbox {B}_\infty = 2$ m. $\hbox {H}_{\mathrm{b}}$ and $\hbox {h}_{\mathrm{b}}$ were computed by propagating Toomey et al.^46^ significant wave height, as explained above, to the depth where $\hbox {h}_{\mathrm{b}} = \hbox {H}_{\mathrm{b}} / \gamma$ holds, with $\gamma = 0.55$. $\hbox {W}_{\mathrm{b}}$ was computed inverting the calibrated displaced Dean profile at $\hbox {z}_{\mathrm{eq}}(\hbox {y}_{\mathrm{s}} + \hbox {W}_{\mathrm{b}},\hbox {t}) = \hbox {z}(\hbox {y}_{\mathrm{s}},\hbox {t}) - \hbox {h}_{\mathrm{b}}(\hbox {t})$.
- For the Fast-Wide Rule: $\hbox {B}_{\mathrm{w}}$ was computed as the mean wave runup, where runup was estimated using the formula of Stockdon^50^ (considering the slope of the EBP at $\hbox {y} = \hbox {y}_{\mathrm{s}}$), $\hbox {R}$ was obtained by inverting the calibrated displaced Dean profile at $\hbox {z}_{\mathrm{eq}}(\hbox {y}_{\mathrm{s}} - \hbox {R,t}) = \hbox {z}(\hbox {y}_{\mathrm{s}},\hbox {t}) + \hbox {B}_{\mathrm{w}}$. Also, $\hbox {h}_{\mathrm{c}}^\prime = \hbox {h}_{\mathrm{c}} = 0.15 \, \hbox {h}_{\mathrm{m}}$, where $\hbox {h}_{\mathrm{m}}$ is the depth where the bed-shear induced by waves reaches its critical value, computed using the formula given by Komar and Miller^51^. This is the way to compute $\hbox {h}_{\mathrm{c}}$ used by Q2Dmorfo, which we also apply here to all analytical models. $\hbox {W}_{\mathrm{c}}$ was computed inverting the calibrated displaced Dean profile at $\hbox {z}_{\mathrm{eq}}(\hbox {y}_{\mathrm{s}} + \hbox {W}_{\mathrm{c}},\hbox {t}) = \hbox {z}(\hbox {y}_{\mathrm{s}},\hbox {t}) - \hbox {h}_{\mathrm{c}}(\hbox {t})$.
- For the Fast-Narrow Rule: all variables were computed like for the Fast-Wide Rule, but $\hbox {R}$ was substituted by $\min (\hbox {R,w}_\chi )$, and then $\hbox {B}$ was re-computed accordingly.
- For the Fast-Exponential Rule: $\hbox {B}_{\mathrm{n}} = 0.5 \hbox {B}_{\mathrm{w}}$, and $\hbox {h}_{\mathrm{c}}^\prime$ and $\hbox {W}_{\mathrm{c}}$ were computed as explained for the Fast-Wide Rule. We assumed $\mu = 1$.
- For the Slow-Wide Rule: ${\widehat{\hbox {B}}}$ was computed as a quantile of the sum of storm surge, astronomical tide and wave runup representative of extreme events, where runup was estimated using the formula of Stockdon^50^ (considering the slope of the EBP at the height reached by the combination of storm surge plus astronomical tide), ${\widehat{\hbox {R}}}$ was obtained by inverting the calibrated displaced Dean profile at $\hbox {z}_{\mathrm{eq}}(\hbox {y}_{\mathrm{s}} - {\widehat{\hbox {R}}},{\mathrm{t}}) = \hbox {z}(\hbox {y}_{\mathrm{s}},\hbox {t}) + {\widehat{\hbox {B}}}$. Also, $\widehat{{\overline{\hbox {h}}}}_{\mathrm{c}}$ was computed as a quantile of the sum of storm surge, astronomical tide and depth of closure, representative of extreme events (with closure depth being estimated like in the Fast-Wide rule case). ${\widehat{\hbox {W}}}_{\mathrm{c}}$ was computed inverting the calibrated displaced Dean profile at $\hbox {z}_{\mathrm{eq}}(\hbox {y}_{\mathrm{s}} + {\widehat{\hbox {W}}}_{\mathrm{c}},\hbox {t}) = \hbox {z}(\hbox {y}_{\mathrm{s}},\hbox {t}) - \widehat{{\overline{\hbox {h}}}}_{\mathrm{c}}(\hbox {t})$. The quantiles considered to compute extreme wave conditions were the 0.99 and the 0.999, which indicate exceedances of about 3.5 days per year and 3.5 days per decade, respectively.
- For the Slow-Narrow Rule: all variables were computed like for the Slow-Wide Rule, but ${\widehat{\hbox {R}}}$ was substituted by $\min ({\widehat{\hbox {R}}}, \hbox {w}_{\chi })$, and then ${\widehat{\hbox {B}}}$ was re-computed accordingly.

## Results and discussion

### Wide beach regime

**Figure 2:**
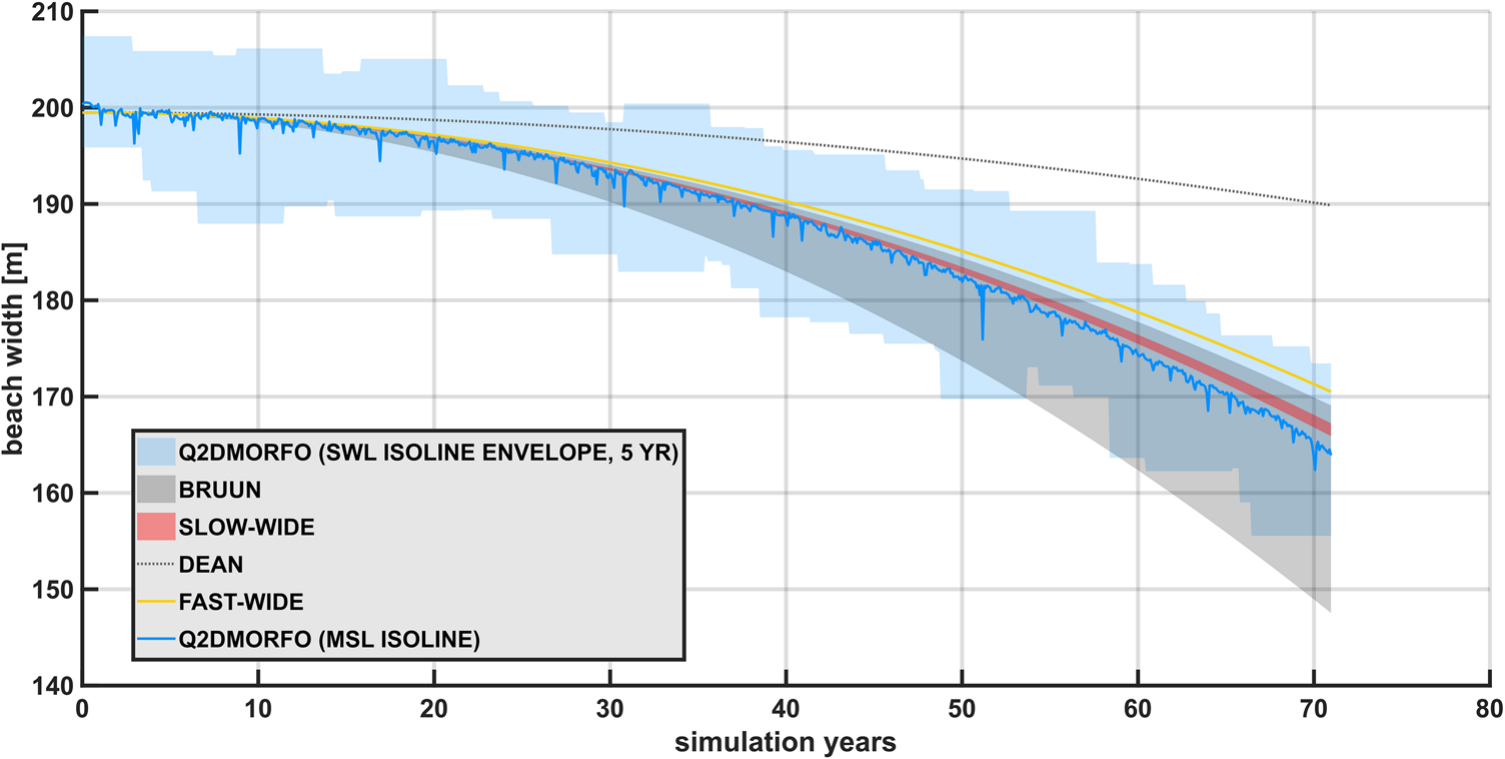
Predicted shoreline recession for an initial emerged beach width of 200 m (wide beach regime), under a parabolic sea-level rise (reaching 1 m at the end of the simulation period) with simultaneous high-frequency forcing. The average shoreline of Q2Dmorfo model and its dispersion are shown by a blue line and a blue shaded area, respectively. They are computed by interpolating the simulated topobathymetry to mean sea level and still-water level, respectively. The shoreline evolution from Ergodic Dean’s Rule is shown with a grey dotted line, that from Fast-Wide Rule is shown in yellow, and Bruun’s Rule is shown as a black shaded area (accounting for all possible emerged active profile boundary). Slow-Wide Rule is indicated by the red shaded area.

Shoreline evolution predicted by each analytical solution and by Q2Dmorfo are presented in Fig. 2, for an initial emerged beach width of 200 m, which is representative of the wide beach regime. The initial shoreline position for the analytical models has been corrected to 199.5 m, to account for Q2Dmorfo initial warm-up. Supplementary Videos also show the evolution of the topo-bathymetry described by Q2Dmorfo in this case.

All the presented models indicate that shoreline change is proportional to changes in mean sea level, but the different models provide different proportionality constants (Fig. 2). Best linear fit between sea-level rise and Q2Dmorfo averaged evolution indicates a slope of 35.9, where the slope of Fast-Wide Rule is 29.8, that of Ergodic Dean’s Rule is 9.9, that of Bruun’s Rule ranges between 31.3 and 53.5, and that of Slow-Wide Rule is between 33.2 and 34.6; this translates into slopes having a value relative to that of Q2Dmorfo of -17.0 % in the case of the Fast-Wide Rule, -72.5 % in the case of Ergodic Dean’s Rule, something between -12.9 % and 48.9 % in the case of Bruun’s Rule, and ranging between -7.6 % and -3.7 % in the case of Slow-Wide Rule. As a result, Root Mean Square Error referred to Q2Dmorfo average evolution for this simulation yields 0.6 m for the Fast-Wide Rule, 11.3 m for Ergodic Dean’s Rule, up to 7.6 m for Bruun’s Rule, and up to 1.3 m for Slow-Wide Rule.

Notice that the light blue shaded area in Fig. 2 does not represent Q2Dmorfo uncertainties, but the range spanned by the instantaneous shoreline within the model numerical simulation (which oscillates rapidly due to storm surges and tides). Thereby, analytical models should not be compared to this Q2Dmorfo dispersion, since they do not describe the variability of instantaneous shoreline. Instead, analytical models should be compared to the time-averaged Q2Dmorfo shoreline (blue line in Fig. 2), which indicates the evolution corresponding to changes in mean sea level, precisely what analytical EBP models intend to describe.

Since Dean model is only valid for periods of constant breaking height, it should not be used directly in the form of Raw Dean’s Rule (Eq. (23)). Ergodic Dean’s Rule (Eq. (25)) should be used instead, which explicitly accounts for changes in wave height. Miller and Dean model (Eq.  (6)) introduces low-pass filtering to Ergodic Dean’s Rule, providing little difference between the two, given the slow changes in mean sea-level rise. Therefore, only Ergodic Dean’s Rule is depicted in Fig. 2, although, in fact, none of these options provide a realistic shoreline retreat.

The difference in shoreline recession predicted by the different models arises from the difference in the slope of active profile they consider. Ergodic Dean’s Rule (or Miller and Dean’s model) uses the shoreline as the onshore boundary for the active profile, and the instantaneous breaking point as the offshore active profile boundary, which results on an active profile width spanning from the shoreline to a breaking point characteristic of average wave conditions in that beach, once the model is integrated. However, instead of considering a breaking depth characteristic of average wave conditions as the active profile height, they consider the sum of that breaking depth and the berm height. This mismatch between the active profile width and height, arising from the consideration of an infinite shoreline slope, results on a very bad shoreline recession prediction for Ergodic Dean’s Rule. Moreover, since there is sediment transport beyond the breaking point^6^, the use of the depth of closure to define the active profile seems more plausible.

Bruun’s Rule considers the depth of closure, instead of the breaking depth, to define the active profile offshore boundary, specifically that corresponding to extreme waves conditions, not the one given by instantaneous wave conditions. Moreover, it does not specify which point between the shoreline and the beach back should be selected as the onshore boundary of the active profile, thereby providing an extremely wide range of predictions around the Q2Dmorfo result. However, it considers the distance from the shoreline and the height above the shoreline up to the same point, thus keeping a consistent relation between active profile width and height, unlike what occurs with the infinite shoreline slope appearing in Dean’s model. As a result, Bruun’s Rule range of possible evolutions overlaps quite well with the shoreline evolution (and its variability) described by Q2Dmorfo.

Fast-Wide Rule also considers the depth of closure, although it uses instantaneous wave conditions, which is equivalent to consider a fixed depth of closure, characteristic of the average wave conditions of the beach, as can be seen during the model integration process. Moreover, it considers the average range of emerged beach topography affected by waves as the onshore active profile boundary, instead of an arbitrary limit, like Bruun’s Rule does. The resulting prediction is much better than that of Dean’s model.

Slow-Wide Rule considers depth of closure and extreme active profile conditions, defined for both the emerged and the submerged parts of the active profile. To define the size of the active profile, it considers the joint effect of high-frequency still-water level and waves, instead of estimating it using only wave forcing, or a morphologic feature. Moreover, it uses the time series of instantaneous depth of closure to derive the depth of closure value characteristic of extreme conditions, instead of a parametrization like that of Birkemeier^49^. This allows to define a range of possible evolutions, corresponding to a range of possible quantiles, to characterize extreme, instead of sticking to an arbitrary value. Overall, it provides the best approximation to the Q2Dmorfo result.

Depending on the curvature of the submerged beach, the decision on the offshore active profile boundary (i.e., related to averaged conditions or extreme conditions) will have more or less impact on the active profile slope: the decision is not important for planar beaches, but is critical for bathymetries with noticeable curvature. Since the considered beach (inspired in Son Bou) presents quite a planar submerged beach, it is not possible to observe how the selection of average or extreme wave conditions affects shoreline recession prediction. However, we expect that for more concave beaches the shoreline evolution indicated the different models will present a higher dispersion.

In a similar fashion, depending on the curvature of the emerged beach, as well as on the difference between the emerged and submerged beach slopes, the selection of the onshore active profile boundary has more or less impact on the overall slope, and thus, on the predicted shoreline recession. In this case, the Bruun’s Rule dispersion on Fig. 2 indicates how the curvature of emerged beach affect shoreline prediction.

To summarize, the obtained results indicate that Slow-Wide Rule is the best estimate for the effects of sea-level rise under the wide beach regime, within EBP theory, since the line corresponding to Slow-Wide Rule is the closest to Q2Dmorfo evolution. In this case, the value of $\hbox {R}$ considered by the Slow-Wide Rule was estimated using the parametrizations presented by Stockdon et al.^50^. The important aspect here is to consider the potential landward range of marine forcing (combination of storm surges, astronomical tides and waves) to transport sediment, instead of calibrating the emerged active profile size using the topo-bathymetry of the beach alone, or a GIS procedure, thus neglecting the wave and storm surge climate.

### Narrow beach regime

**Figure 3:**
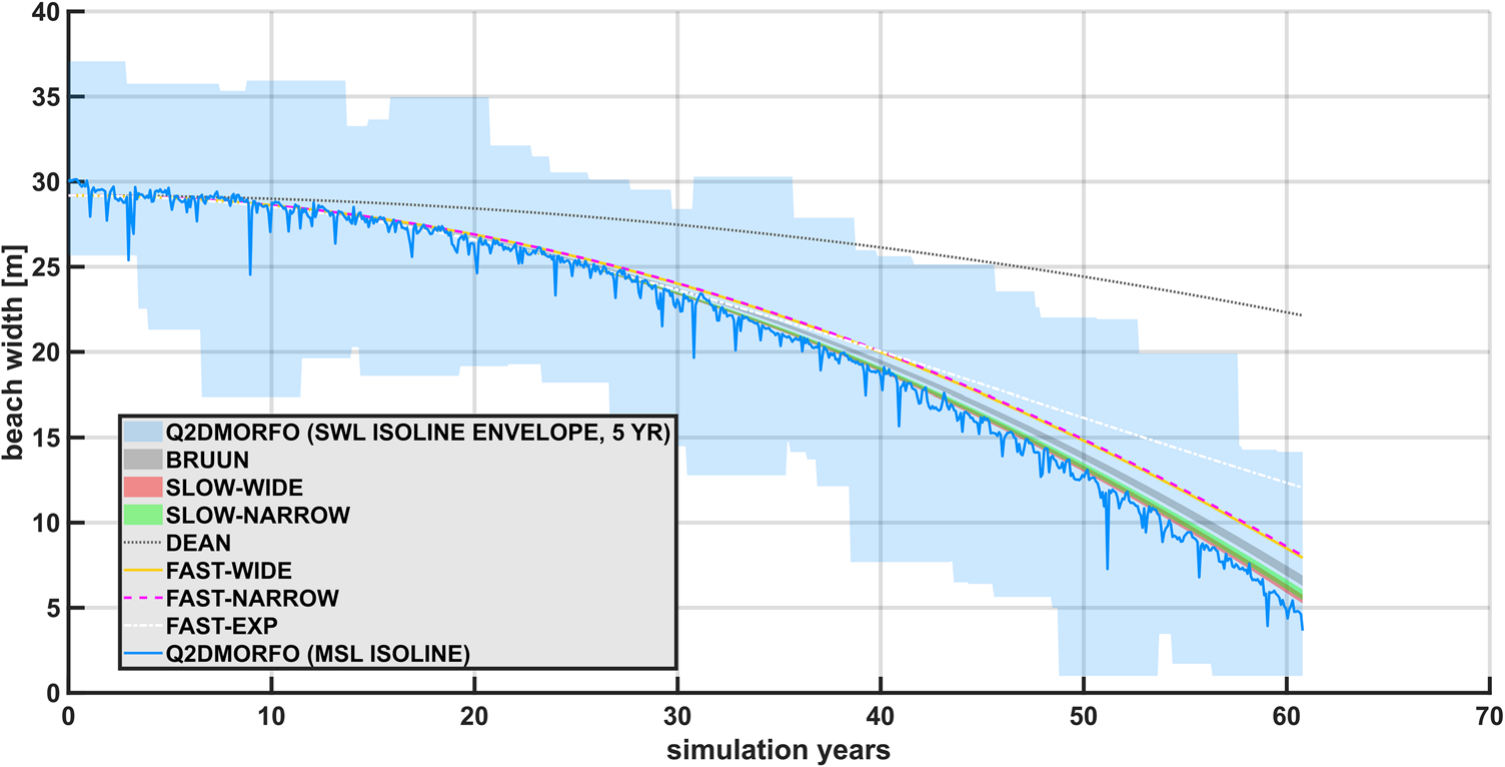
Predicted shoreline recession for an initial emerged beach width of 30 m (narrow beach regime), under a parabolic sea-level rise (reaching 1 m at the end of the simulation period), with simultaneous high-frequency forcing. Fast-Narrow Rule is indicated by the dashed magenta line, while Fast-Exponential Rule is indicated by the dashed white line. Also, Slow-Narrow Rule is depicted by the green shaded area. Refer to Fig. 2 for the meaning of the other lines and shaded areas. Note the Q2Dmorfo simulation ends before completing the 72 years study period, because the instantaneous shoreline (not the time-averaged shown in this figure) arrives to zero, thus ending the simulation.

Shoreline evolution predicted by each model with an initial emerged beach width of 30 m, representative of the narrow beach regime, are presented in Fig. 3, for a parabolic sea-level rise reaching 1 m by the end of the simulation period. The initial shoreline for the analytical models has been located at 29 m, to account for Q2Dmorfo initial warm-up. For the sake of comparison, we have also added the results of the Fast-Wide Rule and Slow-Wide Rule, as well as the Dean and Bruun rules, despite the fact that they do not explicitly consider the beach is narrow. Supplementary Videos also show the evolution of the topo-bathymetry described by Q2Dmorfo in this case.

By construction, Fast-Wide, Narrow-Wide and Ergodic Dean’s Rules indicate the same evolution than for the wide beach regime case presented above, so the same comments apply for them in the narrow beach regime. However, Bruun’s Rule range is quite narrower this time, because the set of possible emerged active profile boundary options is smaller and with very similar slopes, although again Bruun’s Rule range of possible evolutions does not deviate too much from the shoreline evolution described by Q2Dmorfo, which is quite interesting. Fast-Exponential Rule indicates an evolution with significantly less erosion than the others (except Ergodic Dean’s Rule), suggesting that the assumption of a constant berm height in this method is unrealistic. Both Fast-Narrow Rule and Slow-Narrow Rule show a small deviation with respect to their Wide counterparts, indicating less erosion. This is caused by the fact that a smaller emerged part of the EBP must be raised thereby producing less erosion, but the effect is not large because the emerged active profile of the example used is small compared to the submerged active profile.

This time, not all models indicate shoreline changes proportional to changes in mean sea level, so we do not analyze proportionality constants but only Root Mean Square Error, referred to Q2Dmorfo average evolution for this simulation, which evaluates to 7.8 m for Ergodic Dean’s Rule, up to 1.1 m for Bruun’s Rule, 1.6 m for both Fast-Wide and Fast-Narrow, 2.6 m for Fast-Exponential, and up to 0.8 m for both Slow-Wide and Slow-Narrow.

We can observe that Q2Dmorfo indicates a tendency in shoreline evolution different than that predicted by slow-narrow rule, closer to that of slow-wide rule, indicating some inertia to the change from wide to narrow regime, probably caused by not being constrained to a fixed $\hbox {R}$.

Up to this point, it seems that the Slow EBP model is more suited to describe the effects of sea-level rise on shoreline evolution, since their predictions are closer than those of other models to the evolution indicated be Q2Dmorfo. Although Fast EBP model seems less suitable to describe the effects of sea-level rise on shoreline position, it may be helpful to describe the effect of high-frequency forcing, if it is paired with a disequilibrium model.

Additionally, further research is needed to characterize narrow beach shoreline recession induced by sea-level rise using simple analytical models. For instance, exploring how the results obtained change for beaches with a greater ratio between the emerged and submerged parts of the active profile (e.g., low-sloping landscapes) and for beaches whose landward extreme is not fixed. Moreover, extending this study to include sediment exchanges between the active profile and the rest of the beach through mechanisms such as overwash or aeolian transport could provide valuable insights. Finally, a substantial knowledge gap remains concerning the effects of varying EBP shapes according to different incoming wave conditions under the Fast EBP model.

## Conclusions and recommendations

In this work a general equation describing the shoreline evolution in terms of sediment volume balance has been presented, highlighting the importance of using the sediment budget and the time derivative as a tool for obtaining EBP related models, instead of considering two arbitrarily separated EBP states and the difference in forcing values between them. We also propose some corrections to the widely used Dean’s model, namely, inclusion of the emerged active profile into the equations, modification in the coefficients appearing in terms related to the breaking depth (or equivalently, the breaking wave height), and generalization to variable wave conditions by means of integration.

We propose two general EBP models that deal with high-frequency components of forcing either instantaneously or statistically, whose simplified versions were compared to Bruun’s and Dean’s models, as well as a dynamical EBP-based model computing sediment transport (Q2Dmorfo) which was taken as ground truth, for a study case. Under the wide beach regime, i.e., when the action of waves does not reach the backbeach, the rate of shoreline recession differed by -17.0 % using Fast-Wide Rule and between -7.6 % to -3.7 % when using Slow-Wide Rule (which arise from the new EBP models). Instead, the differences are much larger (by -72.5 %) in the case of Ergodic Dean’s Rule (which is the one resulting from Dean’s EBP model), and highly variable (between -12.9 % and 48.9 %) in the case of Bruun’s Rule, always when compared to the rate of shoreline recession given by Q2Dmorfo.

Interestingly, the range of possible shoreline evolutions described by Bruun’s Rule does not deviate excessively from that described by Q2Dmorfo, unlike the results of Dean’s Rule. This indicates the importance of accounting for the part of the active profile within the emerged beach. It also indicates that using the same two active profile points to compute active profile width, height, and slope is essential. However, the uncertainty in the selection of the landward active profile boundary in Bruun’s Rule results in a substantial uncertainty range in shoreline evolution. The model presented reduce this uncertainty by considering the time series of instantaneous forcing to define the size of the active profile (as combination of storm surge, astronomical tide, and either run-up or instantaneous depth of closure). As a result, the new approaches provide a more robust assessment of shoreline retreat under mean sea-level rise conditions than other widely used methods. It is crucial to avoid EBP models which use breaking depth instead of the depth of closure, or that include an infinite slope at the shoreline, or whose solution methods are not based on integrating the corresponding EBP shoreline differential equation (Dean’s approach). Likewise, it is advisable to avoid arbitrary definitions for the boundaries of the active profile (Bruun’s approach).

According to our results, EBP models referred to mean sea level are more suitable to predict the effect of sea-level rise. Although it was not explored in this study, EBP models referred to the instantaneous water level may be useful to predict the variability of shoreline evolution that arises from its high-frequency content, or to predict shoreline evolution in the short term, when paired with a disequilibrium model.

The present study demonstrates that caution is required when using EBP models on narrow beaches. Our model indicates that there may be a change in the regime of shoreline recession induced by sea-level rise in this case, when compared to wider beaches, or to the same beach in a wider state. Unfortunately, we have not been able to observe this change of regime yet, but it is physically clear that narrow beaches have a shorter cross-shore profile to be raised and, thereby, less sediment is needed producing a smaller recession. The last method proposed, Slow Rule, can be used as a generalized tool that allows to compute shoreline recession induced by sea-level rise independently of the beach width, just by means of a look-up table.

The recommendations provided by this study can contribute to a more accurate application of the widely-used EBP models to forecast the shoreline retreat that will be produced by sea-level rise. Despite of their many simplifications and assumptions these models are the only option for large-scale shoreline evolution assessments.

### Supplementary Information

Supplementary Information 1.

Supplementary Information 2.

Supplementary Video 1.

Supplementary Video 2.

Supplementary Video 3.

Supplementary Video 4.

## Data Availability

The datasets used and/or analyzed during the current study available from the corresponding author on reasonable request.
