# Supplementary material for: Shoreline response to sea-level rise according to equilibrium beach profiles
Source: Sci Rep. 2023 Sep 22;13:15789. doi: 10.1038/s41598-023-42672-3 (PMC10517172; doi:10.1038/s41598-023-42672-3)
Supplement: Supplementary file 1 — Supplementary Information 1. [file 41598_2023_42672_MOESM1_ESM.pdf]

Supplementary Note 1: Derivation of the Fast EBP shoreline evolution equation

Pau Luque[1,*], Lluís Gómez-Pujol[2], Francesca Ribas[3], Albert Falqués[3], Marta Marcos[1,4], and Alejandro Orfila[1,]**

[1]Mediterranean Institute for Advanced Studies (IMEDEA), Spanish National Research Council - University of the Balearic Islands (CSIC-UIB), Esporles, Spain.
[2]Earth Sciences Research Group, Department of Biology, University of the Balearic Islands (UIB), Palma, Spain
[3]Department of Physics, Polytechnic University of Catalonia (UPC), Barcelona, Spain
[4]Department of Physics, University of the Balearic Islands (UIB), Palma, Spain
[*]pau.luque@uib.es
[**]alejandro.orfila@csic.es

With the definitions presented in the Section "New EBP Models" of the main text, we can compute the active profile area between the beach bed elevation and a fixed datum as:

$$\mathscr{A} = \int_{y_d}^{y_s} \left(\overline{\eta} + \zeta_s + Z_{wet}(y-y_s;\vec{p})\right) dy + \int_{y_s}^{y_s+W_c} \left(\overline{\eta} + \zeta(y-y_s;\vec{p}) - h(y-y_s;\vec{p})\right) dy. \tag{SN2.1}$$

Then, the time derivative of this area, which corresponds to the time derivative of the amount of sediment contained in the active profile, is given by:

$$\begin{aligned}\frac{d\mathscr{A}}{dt} = \frac{d\overline{\eta}}{dt}\left(W_c + \min(R,w)\right) + \left(\frac{d}{dt}\min(R,w)\right) z_{wet}(y_d) + \frac{dW_c}{dt} z_{sub}(y_c) + \\ + \sum_i \frac{dp_i}{dt}\left(\min(R,w)\frac{\partial \zeta_s}{\partial p_i} + \frac{\partial}{\partial p_i}\int_{y_d}^{y_s} Z_{wet}\, dy + \frac{\partial}{\partial p_i}\int_{y_s}^{y_c} (\zeta - h)\, dy\right).\end{aligned} \tag{SN2.2}$$

By continuity, $d\mathscr{A}/dt$ must be equal to the sum of the net flux of sediment entering the active profile and the sediment sources and sinks occurring within the active profile. We can decompose this as the net sediment flux entering from the landward boundary of the active profile (Q_{dry}), the seaward boundary of the active profile (Q_{deep}), and the alongshore boundaries of the beach region under study (ΔQ_{ls}); plus a term for the net effect of sediment sources and sinks ($\mathscr{S}$), like nourishment's, dredging, endogenous generation of sediment, *etc*. Under these considerations, we can write the sediment budget for the EBP active profile as:

$$\frac{d\mathscr{A}}{dt} - Q_{dry} - Q_{deep} - \Delta Q_{ls} - \mathscr{S} = 0. \tag{SN2.3}$$

Sediment fluxes through the cross-shore boundaries of the active profile (Q_{dry} and Q_{deep}) can be further decomposed into those required by changes in the active profile position and size, and all the other ones (*e.g.*, aeolian transport, river discharges):

$$\begin{aligned} Q_{dry} &= Q'_{dry} - \frac{dy_s}{dt} z_{dry}(y_d) + \left(\frac{d}{dt}\min(R,w)\right) z_{dry}(y_d) \\ Q_{deep} &= Q'_{deep} + \frac{dy_s}{dt} z_{deep}(y_c) + \frac{dW_c}{dt} z_{deep}(y_c). \end{aligned} \tag{SN2.4}$$

Thus, the sediment budget becomes:

$$\begin{aligned}\frac{d\overline{\eta}}{dt}\left(W_c + \min(R,w)\right) + \frac{dy_s}{dt}\left(z_{dry}(y_d) - z_{deep}(y_c)\right) + \\ + \left(\frac{d}{dt}\min(R,w)\right)\left(z_{wet}(y_d) - z_{dry}(y_d)\right) + \frac{dW_c}{dt}\left(z_{sub}(y_c) - z_{deep}(y_c)\right) + \\ + \sum_i \frac{dp_i}{dt}\left(\min(R,w)\frac{\partial \zeta_s}{\partial p_i} + \frac{\partial}{\partial p_i}\int_{y_d}^{y_s} Z_{wet}\, dy + \frac{\partial}{\partial p_i}\int_{y_s}^{y_c} (\zeta - h)\, dy\right) - Q'_{dry} - Q'_{deep} - \Delta Q_{ls} - \mathscr{S} = 0.\end{aligned} \tag{SN2.5}$$

Finally, changes in EBP shoreline are given by:

$$\frac{dy_s}{dt} = -\frac{d\overline{\eta}}{dt}\,\frac{W_c + \min(R,w)}{z_{dry}(y_d) - z_{deep}(y_c)} - \left(\frac{d}{dt}\min(R,w)\right)\frac{z_{wet}(y_d) - z_{dry}(y_d)}{z_{dry}(y_d) - z_{deep}(y_c)} - \frac{dW_c}{dt}\,\frac{z_{sub}(y_c) - z_{deep}(y_c)}{z_{dry}(y_d) - z_{deep}(y_c)} + \\ - \sum_i \frac{dp_i}{dt}\,\frac{\min(R,w)\frac{\partial \zeta_s}{\partial p_i} + \frac{\partial}{\partial p_i}\int_{y_d}^{y_s} Z_{wet}\,dy + \frac{\partial}{\partial p_i}\int_{y_s}^{y_c}(\zeta - h)\,dy}{z_{dry}(y_d) - z_{deep}(y_c)} + \frac{Q'}{z_{dry}(y_d) - z_{deep}(y_c)}. \quad \text{(SN2.6)}$$

where $Q' = Q'_{dry} + Q'_{deep} + \Delta Q_{ls} + \mathscr{S}$ is and effective net sediment flux entering the model domain.
