# Supplementary material for: Shoreline response to sea-level rise according to equilibrium beach profiles
Source: Sci Rep. 2023 Sep 22;13:15789. doi: 10.1038/s41598-023-42672-3 (PMC10517172; doi:10.1038/s41598-023-42672-3)
Supplement: Supplementary file 2 — Supplementary Information 2. [file 41598_2023_42672_MOESM2_ESM.pdf]

Supplementary Note 2: Analytical solution for the narrow beach regime

Pau Luque[1,*], Lluís Gómez-Pujol[2], Francesca Ribas[3], Albert Falqués[3], Marta Marcos[1,4], and Alejandro Orfila[1,]**

[1]Mediterranean Institute for Advanced Studies (IMEDEA), Spanish National Research Council - University of the Balearic Islands (CSIC-UIB), Esporles, Spain.
[2]Earth Sciences Research Group, Department of Biology, University of the Balearic Islands (UIB), Palma, Spain
[3]Department of Applied Physics, Polytechnic University of Catalonia (UPC), Barcelona, Spain
[4]Department of Physics, University of the Balearic Islands (UIB), Palma, Spain
[*]pau.luque@uib.es
[**]alejandro.orfila@csic.es

Statistical considerations

The analysis of shoreline recession caused by sea-level rise necessarily requires statistical tools, since we are not able to predict future magnitudes at the required time scale, and instead we are constrained to characterize them. We require to consider as stochastic the time series of storm surge (Ξ), and the time series of the depth of closure (h_c) as well as the associated active profile width in the submerged beach (W_c). On the contrary, potential future changes in mean sea level (χ) define the shoreline evolution for each studied scenario, and so this must be considered as a known magnitude (regarding the shoreline evolution model, not about the scenario itself), as well as the astronomic tides (Γ). We assume all time series involved are ergodic, so the statistics arising from the ensemble of possible realizations is equivalent to the time statistics of each of the realizations. We also assume they are stationary, so their statistics remain constant through time.

In the following, we will denote the expected value of a time series f(t) as E[f], and the oscillations around this expected value as $\psi_f(t) = f(t) - E[f]$. Thus, a deterministic time series and any realization of an stochastic time series can be written as $E[f] + \psi_f(t)$. Moreover, we define the covariance of two time series f(t) and g(t) (which may be stochastic or not) as:

$$\sigma(f,g) = \frac{1}{t - t_0} \int_{t_0}^{t} \psi_f(\tau)\psi_g(\tau)\, d\tau, \tag{SN1.1}$$

for time intervals $t - t_0$ long enough so ergodicity can be applied. This definition of covariance can be rewritten as:

$$\sigma_\int(f,g)(t,t_0) = \int_{t_0}^{t} \psi_f(\tau)\psi_g(\tau)\, d\tau = \sigma(f,g)(t - t_0) + \psi_{\sigma_\int(f,g)}(t), \tag{SN1.2}$$

where the last term of the equation consists on any term that tends to zero when divided by long enough time $t - t_0$. Finally, we may write the integral of the product of two time series as:

$$\int_{t_0}^{t} f(\tau)\, g(\tau)\, d\tau = E[f] \int_{t_0}^{t} \psi_g(\tau)\, d\tau + E[g] \int_{t_0}^{t} \psi_f(\tau)\, d\tau + E[f]\, E[g]\, (t - t_0) + \sigma_\int(f,g)(t). \tag{SN1.3}$$

Derivation of the analytical model

The solution to EBP shoreline evolution for the new model proposed in the main text under the narrow beach regime is:

$$w(t) = w(t_0)\, e^{-\int_{t_0}^{t} P(\tau)\, d\tau} - \int_{t_0}^{t} e^{-\int_{\tau}^{t} P(s)\, ds} Q(\tau)\, d\tau, \tag{SN1.4}$$

with $P(t) = 1/(B_n + h'_c(t))\, d\overline{\eta}/dt$, $Q(t) = W_c(t)/(B_n + h'_c(t))\, d\overline{\eta}/dt$.

We start by interpreting the integral of P(t). For the term associated to mean sea level (χ), since changes in χ are slower than changes in h'_c by several orders of magnitude, we get the following for long enough integration times (assuming ergodicity and stationarity for the depth of closure):

$$\int_{t_0}^{t} \frac{\frac{d\chi}{dt}(\tau)}{B_n + h'_c(\tau)}\, d\tau \approx \beta \left(\chi(t) - \chi(t_0)\right), \tag{SN1.5}$$

where $\beta = \mathrm{E}\left[[1/(\mathrm{B}+\mathrm{h}_c'(t)) \right]$. For the terms associated to astronomic tides (Γ), and storm surge (Ξ), we use equation SN1.3, and consider that the expected value of the time derivative of astronomic tides (Γ) and storm surges (Ξ) time series are both zero:

$$\int_{t_0}^{t} \frac{\frac{d\Gamma}{dt}(\tau)}{B_n + h_c'(\tau)}\, d\tau \approx \beta\left(\Gamma(t) - \Gamma(t_0)\right) + \sigma\left(p, \frac{d\Gamma}{dt}\right)(t - t_0) + \psi_{\sigma_\int(p, \frac{d\Gamma}{dt})}(t), \tag{SN1.6}$$

$$\int_{t_0}^{t} \frac{\frac{d\Xi}{dt}(\tau)}{B_n + h_c'(\tau)}\, d\tau \approx \beta\left(\Xi(t) - \Xi(t_0)\right) + \sigma\left(p, \frac{d\Xi}{dt}\right)(t - t_0) + \psi_{\sigma_\int(p, \frac{d\Xi}{dt})}(t). \tag{SN1.7}$$

However, since we regard the time series of storm surge (Ξ) as stochastic (with a expected value equal to zero), the first term of the RHS in equation (SN1.7) can also be regarded as a statistical oscillation term. Moreover, since astronomical tides and wave height are independent, the second term of the RHS in equation (SN1.6) is identically zero. Therefore, we may write the integral of P(t) as:

$$\int_{t_0}^{t} P(\tau)\, d\tau = \kappa(t - t_0) + \beta(\chi(t) - \chi(t_0)) + \beta(\Gamma(t) - \Gamma(t_0)) + \psi_{\int P}(t), \tag{SN1.8}$$

where the first three terms represent the expected value of this magnitude, and the last term contains all statistical oscillations. We also defined $\kappa = \sigma(p, d\Xi/dt)$, which is can also be defined as $\kappa = \mathrm{E}\left[d\Xi/dt\, 1/(B_n + h_c') \right]$, since $d\Xi/dt = 0$).

Analogously, the integral of Q(t) is:

$$\int_{t_0}^{t} Q(\tau)\, d\tau = \gamma(t - t_0) + \frac{1}{m_{\mathrm{eff}}}(\chi(t) - \chi(t_0)) + \frac{1}{m_{\mathrm{eff}}}(\Gamma(t) - \Gamma(t_0)) + \psi_{\int Q}(t), \tag{SN1.9}$$

where γ is the covariance $\sigma(q, d\Xi/dt)$ arising from the integration, and where $m_{\mathrm{eff}} = 1/\mathrm{E}\left[W_c(t)/(B_n + h_c'(t)) \right]$ (which acts as an effective slope, if we compare the expression with Bruun's Rule). Consequently, we may write:

$$Q(t) = \gamma + \frac{1}{m_{\mathrm{eff}}}\frac{d\chi}{dt}(t) + \frac{1}{m_{\mathrm{eff}}}\frac{d\Gamma}{dt}(t) + \psi_Q(t). \tag{SN1.10}$$

Using these equivalences for P(t) and Q(t), the general solution of the new model, equation (SN1.4), can be readily rewritten as:

$$\begin{aligned} w(t) = &+ e^{-\psi_{\int P}(t)} w(t_0)\, e^{-(\Omega(t) - \Omega(t_0))} \\ &- e^{-\psi_{\int P}(t)} \int_{t_0}^{t} e^{-(\Omega(t) - \Omega(\tau))} \left(\gamma + \frac{1}{m_{\mathrm{eff}}}\frac{d\chi}{dt}(\tau) + \frac{1}{m_{\mathrm{eff}}}\frac{d\Gamma}{dt}(\tau) \right) e^{\psi_{\int P}(\tau)}\, d\tau \\ &- e^{-\psi_{\int P}(t)} \int_{t_0}^{t} e^{-(\Omega(t) - \Omega(\tau))} \psi_Q(\tau) e^{\psi_{\int P}(\tau)}\, d\tau, \end{aligned} \tag{SN1.11}$$

where we defined $\Omega(t) = \kappa t + \beta\chi(t) + \beta\Gamma(t)$.

Our analytical approximation is based on the expected value of equation (SN1.11). This can not be handled directly, so instead we consider the first term of its Taylor expansion. Since equation (SN1.11) has the form of a ratio (where the denominator is $e^{\psi_{\int P(t)}}$), and since first order Taylor expansion for the expected value of a ratio is the ratio of expected values[1], we can write:

$$w(t) \approx \frac{1}{\mu} w(t_0)\, e^{-(\Omega(t) - \Omega(t_0))} - \frac{1}{\mu}\int_{t_0}^{t} e^{-(\Omega(t) - \Omega(\tau))} \left(\gamma + \frac{1}{m_{\mathrm{eff}}}\frac{d\chi}{dt}(\tau) + \frac{1}{m_{\mathrm{eff}}}\frac{d\Gamma}{dt}(\tau) \right) \mu\, d\tau - \frac{1}{\mu}\int_{t_0}^{t} e^{-(\Omega(t) - \Omega(\tau))} \lambda\, d\tau, \tag{SN1.12}$$

where we have defined $\mu = \mathrm{E}\left[e^{\psi_{\int P}} \right]$, and $\lambda = \sigma\left(\psi_Q, e^{\psi_{\int P}} \right)$, and used the identity $\mathrm{E}[fg] = \mathrm{E}[f]\,\mathrm{E}[g] + \sigma(f, g)$. We rewrite the first integral of equation (SN1.12) in terms of the second one:

$$\begin{aligned} &-\int_{t_0}^{t} e^{-(\Omega(t) - \Omega(\tau))} \left(\gamma + \frac{1}{m_{\mathrm{eff}}}\frac{d\chi}{dt}(\tau) + \frac{1}{m_{\mathrm{eff}}}\frac{d\Gamma}{dt}(\tau) \right) d\tau = \\ &\qquad = -\frac{1}{\beta\, m_{\mathrm{eff}}}\left(1 - e^{-(\Omega(t) - \Omega(t_0))} \right) - \frac{1}{\beta\, m_{\mathrm{eff}}}\left(\gamma\beta\, m_{\mathrm{eff}} - \kappa \right) \int_{t_0}^{t} e^{-(\Omega(t) - \Omega(\tau))}\, d\tau, \end{aligned} \tag{SN1.13}$$

so (SN1.12) becomes:

$$w(t) \approx \frac{w(t_0)}{\mu}\, e^{-(\Omega(t) - \Omega(t_0))} - \frac{1}{\beta\, m_{\mathrm{eff}}}\left(1 - e^{-(\Omega(t) - \Omega(t_0))} \right) - \rho \int_{t_0}^{t} e^{-(\Omega(t) - \Omega(\tau))}\, d\tau, \tag{SN1.14}$$

with $\rho = \gamma - \frac{\kappa}{\beta\, m_{\mathrm{eff}}} + \frac{\lambda}{\mu}$.

If we require the evolution given by equation (SN1.14) not to blow out, then either $\kappa > 0$, or $\kappa = 0$ and $\rho = 0$ (since the integral will increase its magnitude indefinitely under a constant mean sea level if $\kappa = 0$).

Case $\kappa = 0$

If we compute equation (SN1.14) for a case with sea-level rise and a case without it, and then subtract them, we obtain the effects of sea-level rise as:

$$\Delta w_\chi(t) = -\left(\frac{w(t_0)}{\mu} + \frac{1}{\beta\, m_{eff}}\right) e^{-\beta(\Gamma(t)-\Gamma(t_0))}\left(1 - e^{-\beta(\chi(t)-\chi(t_0))}\right), \tag{SN1.15}$$

which describes tendency controlled by the exponential of sea-level rise, modulated by astronomical tides. Since we are only interested in the low-frequency of beach shoreline evolution, we substitute the astronomical tide factor by its expected value, $\varepsilon_- = E\left[e^{-\beta(\Gamma(t)-\Gamma(t_0))}\right]$, obtaining:

$$\Delta w_\chi(t) = -\varepsilon_- \left(\frac{w(t_0)}{\mu} + \frac{1}{\beta\, m_{eff}}\right)\left(1 - e^{-\beta(\chi(t)-\chi(t_0))}\right). \tag{SN1.16}$$

Case $\kappa > 0$

Note the integral of equation (SN1.14) can be written as $\int_{t_0}^{t} e^{-\kappa(t-\tau)-\beta(\chi(t)-\chi(\tau))}\, e^{-\beta(\Gamma(t)-\Gamma(\tau)}\, d\tau$, which can be interpreted as a lowpass filter over the term $e^{-\beta(\Gamma(t)-\Gamma(\tau)}$, due to the convolution with the linear exponential term. Considering this, we can approximate the term of astronomic tides (Γ) in the integrand by its estimated value, $\varepsilon_+ = E\left[e^{\beta(\Gamma(t)-\Gamma(t_0))}\right]$, and then apply integration by parts:

$$\begin{aligned}
-\int_{t_0}^{t} e^{-(\Omega(t)-\Omega(\tau))}\, d\tau \approx -\varepsilon_+ e^{-\beta(\Gamma(t)-\Gamma(t_0))} \int_{t_0}^{t} e^{-\kappa(t-\tau)-\beta(\chi(t)-\chi(\tau))}\, d\tau = \\
= -\frac{\varepsilon_+}{\kappa} e^{-\beta(\Gamma(t)-\Gamma(t_0))}\left(1 - e^{-\kappa(t-t_0)-\beta(\chi(t)-\chi(t_0))}\right) \\
+ \frac{\varepsilon_+}{\kappa} e^{-\beta(\Gamma(t)-\Gamma(t_0))} \int_{t_0}^{t} e^{-\kappa(t-\tau)-\beta(\chi(t)-\chi(\tau))} \beta \frac{d\chi}{dt}(\tau)\, d\tau.
\end{aligned} \tag{SN1.17}$$

Therefore, equation (SN1.14) can be written as:

$$\begin{aligned}
w(t) \approx -\frac{w(t_0)}{\mu} e^{-(\Omega(t)-\Omega(t_0))} - \frac{1}{\beta\, m_{eff}}\left(1 - e^{-(\Omega(t)-\Omega(t_0))}\right) \\
-\rho' e^{-\beta(\Gamma(t)-\Gamma(t_0))}\left(1 - e^{-\kappa(t-t_0)-\beta(\chi(t)-\chi(t_0))}\right) \\
+\rho' e^{-\beta(\Gamma(t)-\Gamma(t_0))} \int_{t_0}^{t} e^{-\kappa(t-\tau)-\beta(\chi(t)-\chi(\tau))} \frac{d\chi}{dt}(\tau)\, d\tau,
\end{aligned} \tag{SN1.18}$$

where we defined $\rho' = \rho\, \varepsilon_+/\kappa$.

Then, to characterize the effects of sea-level rise only, we need to compute this equation for a case with sea-level rise and another without it, and then subtract them. Also, we get rid of all the terms corresponding to transients, *i. e.* those containing a negative linear exponential factor, since they correspond to model spin-up, and their effect would not be there if the reference time t_0 was different. The result is:

$$\Delta w_\chi(t) = \rho' e^{-\beta(\Gamma(t)-\Gamma(t_0))} \int_{t_0}^{t} e^{-\kappa(t-\tau)-\beta(\chi(t)-\chi(\tau))} \frac{d\chi}{dt}(\tau)\, d\tau. \tag{SN1.19}$$

We can simplify even more this equation, for some cases of sea-level rise. If changes in χ do not contain a strong oscillating component, the linear component dominates the negative exponential factor, and so we can substitute $\beta(\chi(t)-\chi(t_0))$ by its first order Taylor approximation, so the argument of the exponential becomes $-\theta(t)(t-\tau)$, where $\theta(t) = \kappa + \beta\, d\chi/dt(t)$. Since now the argument of the exponential is linear with respect to τ, we can substitute $d\chi/dt(\tau)$ by its Taylor series, and integrate by parts, thus obtaining:

$$\begin{aligned}
\int_{t_0}^{t} e^{-\theta(t)(t-\tau)} \sum_{n=1}^{\infty} \chi^{(n)}(t)(-1)^{n-1} \frac{(t-\tau)^{n-1}}{(n-1)!}\, d\tau = \\
= \sum_{n=1}^{\infty} (-1)^{(n-1)} \frac{\chi^{(n)}(t)}{\theta^n(t)} - e^{-\theta(t)(t-t_0)} \sum_{n=1}^{\infty} (-1)^{(n-1)} \frac{\chi^{(n)}(t)}{\theta^n(t)} \sum_{k=0}^{n-1} \theta^k(t) \frac{(t-t_0)^k}{k!},
\end{aligned} \tag{SN1.20}$$

where $\chi^{(n)}(t)$ indicates the nth time derivative of χ. Again, we get rid of the transient terms:

$$\Delta w_{\chi}(t) \approx \rho' e^{-\beta(\Gamma(t)-\Gamma(t_0))} \sum_{n=1}^{\infty} (-1)^{(n-1)} \frac{\chi^{(n)}(t)}{\theta^n(t)}. \tag{SN1.21}$$

Since sea-level rise is a slow changing function, we can get a raw but very insightful approximation by retaining only the first order of this approximation. Moreover, since we are interested only in the slow changes in shoreline position, we can substitute the exponential of tides factor by its mean value (ε_{-}), and also we can generally approximate $\theta(t)$ by κ. In order to refer sea-level rise effects with respect to the current beach condition, we subtract the expression of this approximation particularized for the case of current sea-level rise conditions, obtaining:

$$\Delta w_{\chi}(t) = \rho'' \left(\frac{d\chi}{dt}(t) - \frac{d\chi}{dt}(t_0) \right), \tag{SN1.22}$$

where $\rho'' = \rho' \varepsilon_{-}/\kappa$. This equation indicates that, as a rule of thumb, changes in the emerged beach width (or shoreline position) would be proportional to changes in the first time derivative of sea-level rise.

References

1. Elandt-Johnson, R. C. & Johnson, N. L. *Survival Models and Data Analysis* (John Wiley & Sons, 1980).
